# Ion-Pairing Hydrophilic Interaction Chromatography for Impurity Profiling of Therapeutic Phosphorothioated Oligonucleotides

**DOI:** 10.1021/acs.analchem.5c01407

**Published:** 2025-07-17

**Authors:** Luca Tutiš, Paul D. Ferguson, David Benstead, Adrian Clarke, Carl Heatherington, Chris Gripton, Christina J. Vanhinsbergh, Govert W. Somsen, Andrea F. Gargano

**Affiliations:** † Division of BioAnalytical Chemistry, Department of Chemistry and Pharmaceutical Sciences, Amsterdam Institute of Molecular and Life Sciences, 1190 Vrije Universiteit Amsterdam, de Boelelaan 1085, 1081 HV Amsterdam, The Netherlands; ‡ Centre for Analytical Sciences Amsterdam, Science Park 904, 1098 XH Amsterdam, The Netherlands; § New Modalities and Parenteral Development, Pharmaceutical Technology & Development, AstraZeneca, Macclesfield, Charter Way, Macclesfield SK10 2NA, U.K.; ∥ Chemical Development, Pharmaceutical Technology & Development, AstraZeneca, Macclesfield, Charter Way, Macclesfield SK10 2NA, U.K.; ⊥ Novartis Pharma AG, Fabrikstrasse 2, CH-4056 Basel, Switzerland; # GlaxoSmithKline, Gunnels Wood Road, SG1 2NY Stevenage, U.K.; ∇ Van ’t Hoff Institute for Molecular Sciences, University of Amsterdam, Science Park 904, 1098 XH Amsterdam, The Netherlands

## Abstract

Therapeutic oligonucleotides (ONs) may contain many closely related impurities. Using conventional liquid chromatography (LC) modes, the separation of impurities comprising the same number of nucleotides as the ON product remains a challenge. In this study, we investigated the performance of ion-pairing HILIC (IP-HILIC) as an alternative mass-spectrometry (MS)-compatible LC mode for ON impurity profiling. A fully phosphorothioated, *N*-acetylgalactosamine-conjugated 16-mer antisense ON (full-length product; FLP) served as a model compound, along with shortmer, longmer, PS–PO converted, deaminated (DA) and nonconjugated (NC) products, which are potential impurities. We describe the effect of ion-pairing reagent (IPR) hydrophobicity, eluent pH, and column temperature on IP-HILIC performance, with IPRs reducing the relative contribution of the phosphate moiety on retention, thereby increasing separation selectivity based on the nature of nucleobases and conjugated groups. For a poly­(dT) ladder, the effective peak capacity was reduced from 35 to 22 when introducing triethylamine as IPR; however, improved separations were observed for PS ONs. By employing an eluent containing 25 mM triethylamine acetate (pH 6.3) and a column temperature of 80 °C, IP-HILIC successfully resolved the DA impurities from both the FLP and the NC-FLP. This is noteworthy, as current MS-compatible, one-dimensional LC methods cannot resolve the DA impurity from the FLP, and MS resolution is often insufficient to differentiate the FLP and DA due to a mass difference of less than 1 Da. The proposed IP-HILIC method shows the potential for ON impurity profiling.

## Graphical abstract

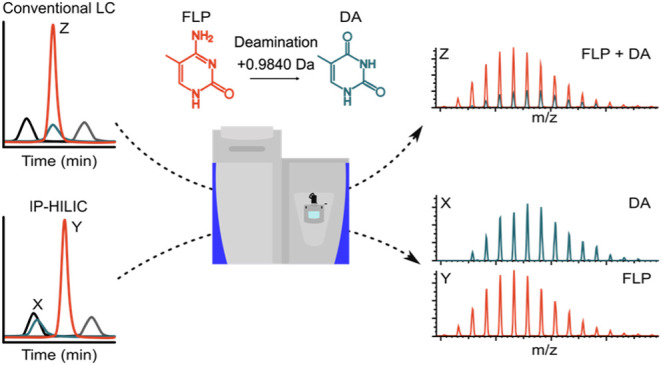

## Introduction

Therapeutic oligonucleotides (ONs) are short strands of DNA or RNA used for the treatment of diseases that cannot be efficiently targeted by other drug modalities.^1^ Antisense oligonucleotides (ASOs) represent an important class with the largest number of approved drugs.^1,2^ ASOs function as gene-expression inhibitors or splicing modulators, aiming to modulate protein expression.^2^ ASOs are typically synthesized by solid-phase supported synthesis (SPSS) and the sequential additions of single nucleotides to a growing chain, employing four reactions per nucleotide: deblocking (detritylation), coupling, oxidation/sulfurization, and capping. During synthesis, side reactions, reaction failures, and degradation reactions can occur.^3^ This commonly results in a full-length product (FLP) comprising a considerable number of closely related impurities, including shorter and longer sequences (shortmers and longmers, respectively). Moreover, to improve the pharmacokinetic properties of ONs, chemical modifications are often introduced,^4^ such as 2′-substituted riboses, methylated bases, or sulfurization of the phosphate group. The latter results in phosphorothioated (PS) groups, which significantly reduce enzymatic (nuclease) degradation in the body compared to phosphodiester ONs,^5^ but also increases sample complexity as each PS introduces a chiral center, leading to 2*^n^* diastereomers for an ON with *n* PS groups.^6^ Additionally, to aid targeted drug delivery, the ON can be covalently conjugated with a ligand to increase cellular uptake, e.g., a carbohydrate moiety, such as *N*-acetylgalactosamine (GalNAc), which facilitates receptor-mediated uptake of ONs specifically to hepatocytes in the liver.^7^ As of 2025, there are at least nine candidate ONs in clinical trials that exploit GalNAc conjugation.^8^ Clearly, the molecular complexity of the current ONs poses a significant analytical challenge.

In order to ensure quality, efficacy, and safety of therapeutic ON products, impurity profiling is imperative. Ion-pairing reversed-phase liquid chromatography (IP-RPLC) is the predominant analytical technique used for this purpose in the ON industry.^9^ In IP-RPLC, positively charged ion-pair reagents (IPRs) are used to increase the retention of the negatively charged ONs on the hydrophobic stationary phase, leading to a length-based separation, as every additional nucleotide increases the apparent hydrophobicity. Impurities that comprise the same number of nucleotides as FLP are, therefore, typically not or poorly resolved. Separations of sequence variants have been reported,^10^ but usually the sequence differs significantly. In this respect, the deaminated product, a sequence variant impurity that only differs on one position and could cause off-target effects,^11^ is one of the most challenging impurities to separate and quantify. During deamination, a cytosine or 5-methylcytosine (MeC) of the ON is converted to uracil or thymine (T), respectively, corresponding to a loss of ammonia and a gain of water.^3^ The molecular mass of this impurity differs by less than 1 Da from that of the FLP, resulting in overlapping isotope patterns in mass spectrometry (MS), hindering their reliable distinction. So far, three approaches have been described to quantify the deamination impurities in ONs. First, weak anion exchange chromatography (WAX) employing a high-pH eluent was used to resolve the deaminated products from the FLP.^12^ The second, MS-based approach focused on statistical modeling of the subtle changes in the isotope pattern when the impurity coelutes with the FLP,^13^ and recently, supercritical fluid chromatography has been reported to separate DA impurities from the FLP.^14^ However, these approaches have limitations, such as MS incompatibility of the WAX method due to the high concentrations of involatile salts used in the eluent, the need for high-resolution and sensitive MS, which is not prevalent in a quality control setting, or it has not been applied to PS ONs. Besides the aforementioned deamination impurity, deamination of adenine and guanine (G) to hypoxanthine and xanthine, respectively, can also occur, but these processes are much slower.

Alongside IP-RPLC, strong anion exchange chromatography (AEX) and hydrophilic interaction chromatography (HILIC) have been used for impurity profiling of ON products.^15,16^ AEX and HILIC methods have a different separation selectivity compared to IP-RPLC, enabling, besides the separation of shortmer and longmer impurities, the separation of oxidized phosphorothioate (PO) impurities from phosphorothioated FLPs. This difference is related to the difference in acidity (PS has a lower p*K*_a_) and hydrophilicity (PS is more hydrophobic) of the PO impurity, which are factors that contribute to the separation of the methods, respectively.^17–20^ However, separation of deaminated products also remains challenging with these LC modes. In the present work, we studied the usefulness of IP-HILIC as an alternative mode for the impurity profiling of phosphorothioated ONs. IP-HILIC has been shown to be highly useful for glycoprotein analysis, where negatively charged IPRs (usually trifluoroacetic acid) shield positive charges of the protein backbone, thereby enhancing the contribution of glycosylation to retention and providing unique resolution of protein glycoforms.^21–26^ The application of IP-HILIC to ON analysis has thus far been quite limited and has not been extensively studied.^27–29^ Gong has demonstrated the potential of IP-HILIC to resolve longmer impurities as well as some chemically modified ONs.^30^ In this research, we investigated the use of IP-HILIC for the impurity profiling of therapeutic ONs using a representative GalNAc-conjugated, fully phosphorothioated 16-mer ASO (FLP) as a model compound. We hypothesized that the addition of positively charged IPRs to the HILIC eluent reduces the contribution of highly negatively charged phosphate backbone of ONs on retention, thereby increasing the HILIC selectivity toward the composition of nucleobases and presence of GalNAc conjugation. We especially aimed at resolving more closely related impurities comprising the same number of nucleotides as the FLP. For that, we systematically explored the effect of IPR physicochemical properties, hydrophobicity and concentration, column temperature, and eluent pH on ON retention and chromatographic performance. The potential of IP-HILIC for impurity profiling was evaluated by analyzing the shortmer, longmer, PS–PO converted, deaminated, and nonconjugated (NC) products of the FLP, and applying IP-HILIC-MS to FLP containing low levels of added impurities.

## Experimental Section

### Chemicals and Reagents

Ammonium acetate (AA) (BioXtra, ≥98%), diethylamine (DEA) (≥99.5%), triethylamine (TEA) (≥99.5%), tripropylamine (TPA) (≥98%), tributylamine (TBuA) (≥99.0% GC), and ethylenediaminetetraacetic acid (EDTA) (ACS reagent, 99.4–100.6%, powder) were obtained from Sigma-Aldrich (Darmstadt, Germany). Glacial acetic acid (Suprapure, 100%) and a 25% ammonia solution (ISO, Reag. Ph Eur) were obtained from Supelco (Darmstadt, Germany). Acetonitrile (ACN) (HPLC-R and LC-MS grade) was obtained from Biosolve (Valkenswaard, The Netherlands). A poly­(dT) ladder MassPREP Oligonucleotide standard was obtained from Waters (Milford, MA, USA). All water used for the solvent and eluents was obtained from a Milli-Q Purification System from Millipore (Merck Millipore, Burlington, MA, USA).

### ON Samples

A model GalNAc-conjugated 16-mer ASO (FLP) and related impurities were provided by AstraZeneca (Table 1). The full sequences can be found in Table S1. All the GalNAc-conjugates are fully phosphorothioated and composed of adenosine (A), 5-methylcytosine (MeC), guanine (G), thymine (T), and 2-deoxyribose (d). The related impurities were a singly deaminated FLP (MeC to T) at position 2 from the 5′ side (deamination, DA), an FLP with one PS–PO conversion (PO), a longmer comprising an additional dG at the 5′ side (*N* + 1), a shortmer having one dG less at the 5′ side (*N* – 1), and an FLP for which the dGs and dTs were switched by dTs and dGs, respectively (Switch impurity, nonisobaric). Moreover, a nonconjugated (NC) and NC singly deaminated (DA-NC) impurity at the same position as DA were provided by GSK. These compounds have the same sequence as the FLP and DA, respectively, but only lack the GalNAc. An 8-mer siRNA comprising 2 PS linkages and 2′-modifications (2′-fluoro (f) and 2′-O-methylation (o)) was provided by Novartis. All ONs were dissolved in ACN–water (75:25, v/v) at a concentration of 1 mg/mL for HILIC measurements and in 100% water for AEX and IP-RPLC measurements. The poly­(dT) ladder consisting of 15-, 20-, 25-, 30-, and 35-mer was dissolved in 540 μL of ACN–water (75:25, v/v).

**Table 1:** Model PS ON Names, Impurities with the Affected Nucleotide Position (*N*) from the 3′ Side, and Average Molecular Mass

name	impurity	avg. mol. mass (Da)
FLP	NA^a^	6793.98
DA	N15: dMeC to dT	6794.96
PO	N8/9: PS to PO	6777.92
*N* + 1	N17: +dG	7139.25
*N* – 1	N16: −dG	6448.71
Switch	dTs switched with dGs	6819.00
NC	–GalNAc	5162.25
DA-NC	–GalNAc, N2: dMeC to dT	5163.23
8-mer	NA	2601.80

a NA: not applicable.

A solution of the FLP (450 μg/mL) with the *N* – 1, *N* + 1, PO, and DA-NC each at 10 μg/mL was made by mixing the 1 mg/mL standard solutions in the appropriate ratios. Additionally, a mixture of NC (450 μg/mL) and DA-NC (10 μg/mL) was made. For IP-RPLC measurements, the FLP and each impurity in the mixtures were 900 and 20 μg/mL, respectively.

### LC-UV and LC-MS Instrumentation

For IP-HILIC and IP-RPLC, an Agilent 1290 Infinity II UHPLC system was used, consisting of a 1290 binary pump (G7120A) containing a V35 jet weaver mixer and a 1290 Multisampler (G7167B) with draw and eject speeds set to 100 and 400 μL/min, respectively. It contained a 1290 MCT (G7116B) with InfinityLab Quick-Connect heat exchangers (P/N: G7116-60015) and a 1290 DAD FS detector (G7117A) containing a Max-Light Cartridge Cell (10 mm; *V* = 1 μL). UV absorbance was measured at 260.0 nm with a bandwidth of 4.0 nm at 10 Hz. For the AEX measurements, the same modules were used, except for the pump, which was a 1260 Bioinert quaternary pump (G5654A).

LC-UV-MS measurements were performed on a 1290 Agilent infinity II UHPLC system, consisting of a binary pump (G4220A) and a HiP Sampler (G4226A). Moreover, it contained a column compartment (G1316C) and a DAD detector (G4212A). The LC system was hyphenated to a Thermo Q-Exactive Orbitrap MS equipped with a heated electrospray ionization (ESI) source set to the negative mode. The following parameters were used: ISCID, 20 eV; capillary temperature, 275 °C; spray voltage, 3.30 kV; S-lens RF, 50; sheath gas, auxiliary gas, and sweep gas flow, 60, 10, and 0 units, respectively. Full scan experiments were performed with a resolution of 140.000, 4 microscans, AGC target of 1 × 10^6^, maximum IT of 200 ms, and scan range of 400–2500 *m*/*z*.

### IP-RPLC and AEX

The IP-RPLC conditions utilized were essentially those according to Rentel et al.^9^ Briefly, the eluent was generated by mixing 5 mM TBuAA in ACN–water (10:90, v/v) (A) and 5 mM TBuAA in ACN–water (80:20, v/v) (B) at pH 7, both containing 1 μM EDTA. An Acquity UPLC BEH C18 column (2.1 mm × 150 mm, 1.7 μm *d*_p_, 130 Å) (P/N: 186002353) from Waters was used. The gradient program started with a 1 min hold at 40% B, followed by a linear increase of 40–60% B in 20 min. The eluent was held at 60% B for 2 min, followed by a flush to 100% B for 2 min. The flow rate was 0.2 mL/min, and the column oven temperature was 60 °C.

For AEX, a TSKgel DNA-STAT column (4.6 mm × 100 mm, 5 μm *d*_p_) (P/N: 0021962) from TOSOH Bioscience was used. The eluent was generated by mixing 20 mM Tris–HCl (pH 8) in ACN–water (10:90, v/v) (A) and 20 mM Tris–HCl (pH 8) with 2 M NaCl in ACN–water (10:90, v/v). The gradient program started with a 1 min hold at 35% B, followed by a linear increase of 35–55% B in 20 min and a hold of 2 min at 55% B. The flow rate was 0.4 mL/min, and the column oven temperature was 25 °C.

### HILIC and IP-HILIC

All (IP-)­HILIC experiments were performed on an Acquity UPLC BEH amide column (2.1 mm × 150 mm, 1.7 μm *d*_p_, 130 Å) (P/N: 186004802) except for the evaluation of triethylamine acetate (TEAA) concentration (Figure 4), which was obtained using a Premier BEH Amide with the same column dimensions. The IPRs DEA, TEA, TPA, and TBuA, as well as AA were tested at a concentration of 15 mM in eluent A and B, where the pH of the aqueous part of the eluents was adjusted with acetic acid or ammonia to pH 7 for the IPRs and AA, respectively, resulting in the alkylamine salts (e.g., diethylamine acetate (DEAA)). Eluent A consisted of ACN–water (20:80, v/v), and eluent B consisted of ACN–water (90:10, v/v). The gradient program started with a 1 min hold at 100% B, followed by a linear decrease of B to 30% from 1 to 50 min, and ending with a 3 min hold at 30% B. The flow rate was 0.2 mL/min. For all ON samples, the injection volume was 1 μL unless specified differently. The poly­(dT) ladder solution was injected at a volume of 5 or 10 μL. The column oven temperature was 45 °C.

For the evaluation of the effect of TEA concentration (5–100 mM), a stock solution of 1 M TEAA at pH 7 in water was used. Eluent A was obtained by diluting the TEAA solution to the desired concentration using Milli-Q water. To prepare eluent B, we added different volumes of TEAA stock solution (0.5–10% v/v) to ACN to achieve the desired IP concentration. This resulted in a change of water content in B; to adjust for this the gradient started with a 1 min hold at different % B depending on the ion concentration: 81, 82, 83, 85 (5, 10, 25, 50 mM, respectively, to normalize to the 100 mM TEAA gradient of 90–60% MPB); followed by a linear decrease to 54, 55, 56, 57% B from 1 to 21 min, and a hold at the highest % B for 2 min. The flow rate was 0.2 mL/min. In the analysis of the effect of pH (4.8–9) of the eluent and column temperature (5–80 °C), eluent A consisted of Milli-Q water with TEAA and eluent B consisted of ACN with TEAA. The gradient started with a 1 min hold at 80% B, followed by a linear decrease to 66.7% B from 1 to 21 min, and a hold at 66.7% B for 2 min. The flow rate was 0.2 mL/min. Final conditions for the analysis of mixtures of FLP with impurities were 25 mM TEA in both eluents A and B, an eluent pH of 6.3, and column temperature of 80 °C, applying an 80–66.7% B gradient as mentioned above.

### Data Processing

LC-UV data was processed using MATLAB 2024b. Peak widths were extracted from Agilent Openlab CDS software (version 2.7). MS data was processed with Thermo Scientific Freestyle version 1.8.65.0. Mass deconvolution was performed with UniDec.^31^ The MS data presented are available at the following Zenodo Repository link: https://zenodo.org/records/14927266. Log *P* and p*K*_a_ values were obtained from PubChem.^32^ Peak capacities (*n*_c_) were calculated using eq 1:

1 $$n_{c}=\frac{t_{G}}{1.7\cdot {\mathrm{w̅}}_{0.5}}+1$$

where *t*_G_ is the gradient time and *w̅*_0.5_ is the average peak with at half-height.

## Results and Discussion

In HILIC, the retention of analytes is primarily based on polar interactions with the stationary phase, largely originating from the hydrophilic moieties of the retained molecule. HILIC elution is typically achieved by using a gradient starting with high amounts of ACN and gradually increasing the water percentage, where the (buffer) salt concentration is kept constant throughout the run and prepared at a specific pH. HILIC is MS-compatible and increasingly used for ON characterization as its selectivity differs slightly from IP-RPLC, and in principle, no IPRs are needed in the eluent.^18–20,33–35^ Given the highly hydrophilic nature of ONs, the percentage of water used for elution ranges between 30 and 70%, suggesting a limited contribution of hydrophilic partitioning to the HILIC retention mechanism. The retention of ONs is most probably driven by hydrogen bonding and ionic interactions. The highly polar, negatively charged phosphate groups of ONs add strongly to retention, allowing separation of shortmer, longmer, and PO impurities from a phosphorothioated FLP on amide-based stationary phases. The resulting separation is relatively similar to IP-RPLC, where the number of phosphate moieties on the ON (that interact with IPRs in IP-RPLC) drives the retention.

In our work, we aimed to develop an HILIC method that can resolve ONs based on their nucleobase composition and chemical modifications by reducing the influence of the phosphate backbone on retention. To accomplish this, we introduced positively charged IPRs into the mobile phase (IP-HILIC). Cationic IPRs are known to interact with the negatively charged ONs, neutralizing their charge and altering their interaction with the HILIC stationary phase. Under these conditions, the HILIC retention will be less dependent on the phosphate backbone (and thus the number of nucleotides) and, resultingly, will be largely driven by the nucleobase composition and conjugated groups of the ON, unlike IP-RPLC, where the IPRs significantly increase retention due to its hydrophobicity. This process is schematically visualized in Figure 1 and more extensively in Figure S1.

**Figure 1:**
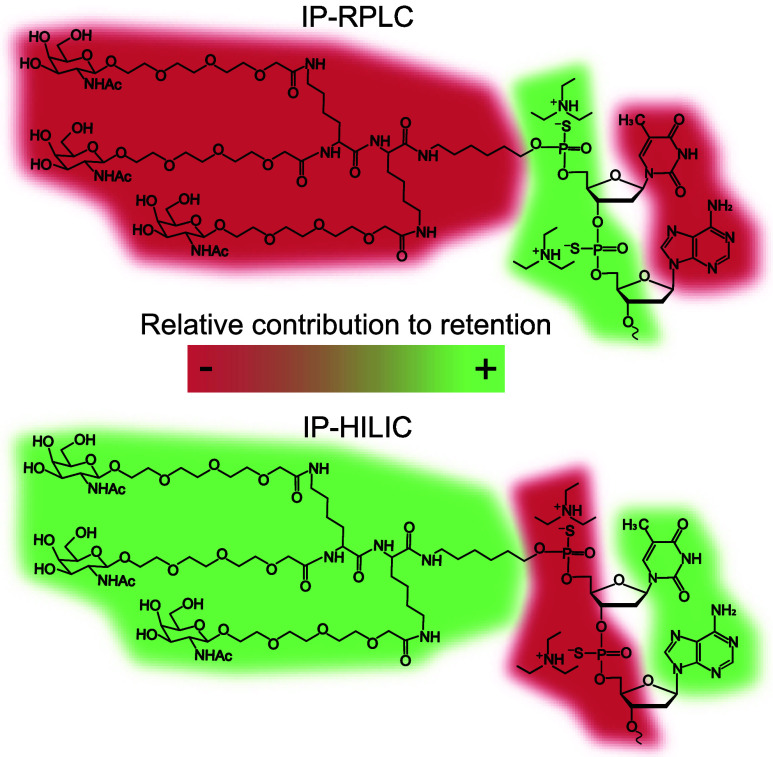
Schematic representation of the effect of IPRs on the eluent for ONs separation in RPLC and HILIC.

### IP-HILIC: Effect of Ion-Pairing Reagent Type on Retention and Selectivity, and Comparison with IP-RPLC and AEX

To assess the effect of ion-pairing on ON retention in HILIC, IPRs of different physicochemical properties and hydrophobicity (DEAA, TEAA, TPAA, and TBuAA) were tested by analyzing a poly­(dT) ladder applying a linear gradient from 90% ACN to 41% ACN (i.e., 100 to 30% B) on a BEH amide column. This sample does not contain any PS modifications and therefore no diastereomers, allowing us to evaluate the effect of IPRs in the eluent on ON peak widths and separation resolution. Using a concentration of 15 mM of IPR in the eluent, (partial) separation of the five ONs was obtained (Figure 2), where the HILIC retention of the poly­(dT) ONs decreased with increasing IPR hydrophobicity. Compared with an eluent containing no IPR but 15 mM AA instead, a retention-time reduction of approximately 30 min was observed for the 15-mer poly­(dT) when using TBuAA in the eluent. The interaction of the positively charged IPRs with the negatively charged phosphate groups decreases the apparent hydrophilicity of the ONs and, thus, their retention. Along these lines, the retention increases with ON size as every additional nucleotide adds a phosphate group, increasing the overall hydrophilicity of the ON.

**Figure 2:**
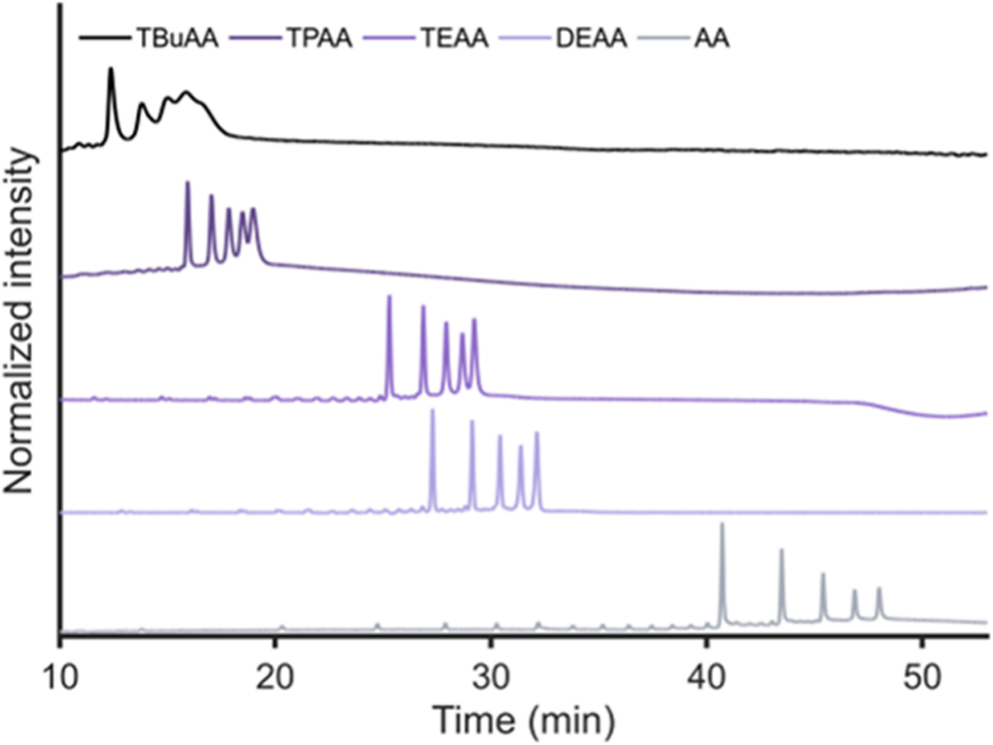
HILIC-UV of a poly­(dT) ladder comprising 15-, 20-, 25-, 30-, and 35-mer using an eluent containing 15 mM of AA, DEA, TEA, TPA, or TBuA (pH 7) using a linear gradient from 100 to 30% B in 50 min on a BEH amide column at 45 °C, with eluents A and B consisting of 90:10 and 20:80 ACN–water (v/v), respectively. UV absorbance detection was performed at 260 nm, and baselines were subtracted using blank measurements (see Figure S2). For other conditions, see the Experimental Section.

In addition to affecting the retention of the poly­(dT) ONs, the IPRs also had noticeable differences in peak widths and resolution. The mobile phase with 15 mM AA (no IPR) resulted in the highest resolution and smallest peak widths, and thus the most favorable peak capacity (*n*_c_) of 258 (Table S2). Adding IPRs to the eluent increased peak widths significantly and decreased the resolution of the poly­(dT) ONs, with the adverse effect becoming more pronounced as the IPR hydrophobicity increases. Peak capacities of 243, 201, and 134 were obtained for DEAA, TEAA, and TPAA, respectively (a similar trend is observed for the effective peak capacities (i.e., the time between the first and last eluting peak as *t*_G_): 35, 22, 14, and 7 for AA, DEAA, TEAA, and TPAA, respectively). Peak capacity values could not be calculated for TBuAA as coelution of the poly­(dT) ONs prevented the determination of peak widths. But clearly, the peak widths obtained with TBuAA are broader than those obtained with the other IPRs (Figure 2). In gradient elution, peak widths should be relatively consistent, independent of the retention time. However, peaks eluting close to *t*_0_ might experience peak broadening due to the injection (lack of on-column focusing). As the ONs with TBuAA in the eluent are retained for over 10 min, this effect is not expected to be a significant contributor to the observed peak widths. Interestingly, the effect of IPR hydrophobicity on IP-HILIC separations is the opposite of IP-RPLC, for which an increase in IPR hydrophobicity results in higher peak capacities.^10^ No PS modifications, and thus no diastereomers, are present in the poly­(dT) sample, and therefore, the reduction in IP-HILIC separation performance can be attributed solely to the influence of the IPRs on ON retention. It may be related to secondary hydrophobic interactions disturbing the HILIC separation. These could take place in two different ways: (i) ON-IPR interactions decrease the apparent hydrophilicity, leading to decreased interactions between the ON and HILIC stationary phase. Therefore, an increase in hydrophobicity of the IPR increases the disturbance of the polar interactions of the ONs with the HILIC stationary phase, leading to broader peaks; (ii) The positively charged IPR can interact with the hydrophilic stationary phase, reducing its hydrophilicity, where the longer the alkyl chain of the IPR is, the stronger the secondary hydrophobic interaction may be.

While the introduction of IPRs to the eluent reduced separation performance, the results suggested that IPRs mitigate interactions with the HILIC stationary phase due to a decreased influence of the phosphate moieties on retention. This prompted us to investigate potential selectivity changes and benefits.

We tested the effect of IPR hydrophobicity on the IP-HILIC impurity profile using a fully PS 16-mer ASO conjugated with GalNAc as a model PS ON pharmaceutical along with several reference impurities that are commonly encountered after synthesis (see Table 1). Notably, each PS ON species comprises over 10 000 potential diastereomers. These compounds were used to test the influence of IPRs on the HILIC retention of the FLP (i), degree of diastereomer separation (ii), the separation between the FLP and the impurities comprising different (iii) and the same number (iv) of nucleotides, and the separation of the FLP from its non-GalNAc-conjugated impurity (v).

The (IP-)­HILIC chromatograms obtained for the PS ONs without and with IPRs added to the eluent are depicted in Figures S3 and S4. Using the same eluent compositions and linear gradient as those used for the poly­(dT) ladder, the PS ONs eluted earlier relative to the poly­(dT) 15-mer in all cases, even though the PS ONs contained more negative charges. Most probably, phosphorothioation creates more ON net hydrophobicity than phosphodiester phosphate bonds due to P=O being more electronegative than P=S. The effect of the IPRs on the peak widths is shown in Figure S5 and Tables S3 and S4, where the replacement of AA by IPRs leads to a decrease in peak widths up to TEAA. A further increase of IPR hydrophobicity presented increased peak widths compared to TEAA. The PS ONs contain diastereomers that could partially be resolved and can be structurally affected by IPRs in the eluent. The decrease in peak width observed when going from AA to TEAA suggests that diastereomer separation is decreased when the hydrophobicity of the IPR is increased, which is also observed for IP-RPLC.^6^ This was further confirmed when analyzing an 8-mer ON with 2 PS linkages and 4 possible diastereomers, which clearly showed decreased diastereomer separation with IPRs of increasing hydrophobicity in the eluent (Figure S6). We concluded that DEAA and TEAA provided a good compromise between peak broadening due to the IPR’s effect on HILIC retention and due to diastereomer separation.

When AA is replaced by an IPR in the eluent, the separation selectivity is also clearly affected, as is visible in Figures S3 and S4. For each additive, the resolution (*R*_s_) between the FLP and the respective impurity was calculated and is plotted in Figure 3, where a negative or positive *R*_s_ value indicates elution before or after the FLP, respectively. The *N* – 1 and *N* + 1 impurities are baseline separated from the FLP using AA (no IPR) in the eluent, and the resolution decreases when AA is replaced by IPRs. This effect is similar to the decreased resolution of the poly­(dT) ONs, where the separation between ONs comprising different number of nucleotides decreased.

**Figure 3:**
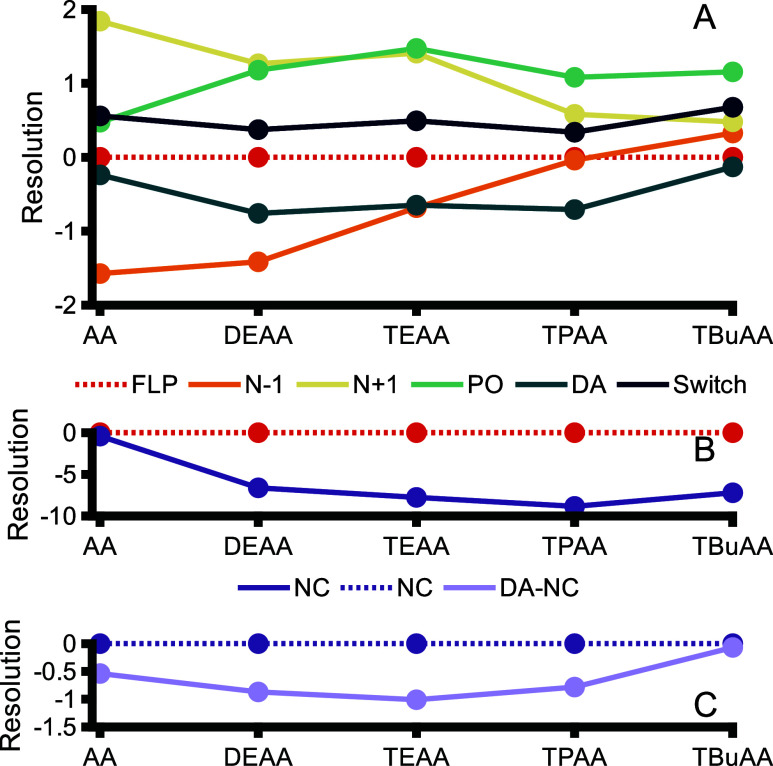
Resolutions obtained during IP-HILIC of the model FLP (fully PS 16-mer ASO conjugated with GalNAc) and related impurities using the same experimental conditions as described for Figure 2. (A) Resolution of *N* – 1, *N* + 1, PO, DA, and Switch with respect to FLP, (B) resolution of NC with respect to FLP, and (C) resolution of DA-NC with respect to NC.

However, compared to using AA, the separation between DA and FLP is improved when DEAA, TEAA, or TPAA are added to the eluent. ON deamination involves the conversion of 5-methylcytosine (log *P*, −0.8) to thymine (log *P*, −0.6), resulting in a decrease of the hydrophilicity of the nucleobase. We suggest that when using AA in the eluent (HILIC), this minor difference in hydrophilicity only slightly affects resolution, as retention is dominated mainly by the thiophosphate groups. However, when IPRs are added to the eluent, the polar interactions of the phosphate backbone are diminished and the relative contribution of the nucleobases on retention is increased. The DA impurity is slightly less hydrophilic and elutes before the FLP, which is also in line with reported results for HILIC of nucleosides,^36^ whereas for the WAX method, DA elutes after the FLP due to the additional charge on DA.^12^ For the nonconjugated (NC) impurity, the resolution with its deaminated form (DA-NC) is even better as compared with the GalNAc-conjugated FLP (see Figure 3C, resolution of −1.00 and −0.65 for TEAA, respectively), suggesting that the GalNAc group, which is close to the deamination site, reduces DA-FLP resolution. This may be due to its contribution to the FLP hydrophilicity or because it causes steric hindrance, affecting interactions. For all tested conditions, DA-NC was better resolved from NC compared to the separation of DA from FLP. Overall, TEAA offered the best results in terms of resolving DA impurities.

The addition of IPRs to the eluent also significantly improved the resolution between FLP and NC (Figure 3B). With AA in the eluent, NC coelutes with the FLP. In contrast, by adding IPRs to the eluent, a resolution of 5 or higher can be obtained, indicating that the relative contribution of the GalNAc on HILIC retention is also increased by IPRs. Interestingly, the PO impurity is better separated from the FLP when AA is replaced by smaller IPRs. However, increasing the IPR hydrophobicity further than TEAA decreases resolution between the PO impurity and the FLP. A similar separation was observed by Gong.^30^ The ability to separate the PO impurity from the FLP in the presence of IPRs might be related to differences in acidity between the PO and PS groups affecting interaction strength between ON and IPR. Also, steric effects may play a role. An ON for which the G nucleotides were switched with T nucleotides (Switch impurity) comprising the same number of nucleotides as the FLP was also measured by using different IPRs. As can be seen in Figure 3A, Switch is partly separated from the FLP with most separation using TBuAA. Overall, from our study of the influence of IPR type on ON impurity separation, we concluded that TEAA provides the most favorable IP-HILIC conditions for impurity profiling, with DEAA as a close second, as it resulted in the smallest peak widths and a slightly better separation of deamination impurities compared to DEAA, which are the most challenging to identify.

We also compared the IP-HILIC method employing TEAA in the eluent with the IP-RPLC and AEX methods using standard conditions. All methods provide separation of *N* – 1, *N* + 1, and NC impurities from the FLP, while IP-HILIC and AEX also separated the PO impurity (Figures S7–S10). However, using IP-RPLC and AEX, the DA impurity fully coeluted with the FLP, whereas IP-HILIC separates DA from the FLP, highlighting the complementary separation selectivity and benefits of IP-HILIC.

### Separation Optimization

To further improve the separation of the deamination and other impurities from the FLP, the roles of concentration of the TEA in the eluent, the eluent pH, and column temperatures were systematically investigated.

### Ion-Pair Reagent Concentration of the Eluent

To assess the effect of IPR concentration in the eluent on the separation of PS ONs in IP-HILIC, TEA concentrations of 5, 10, 25, 50, and 100 mM were tested (Figures 4 and S11). The temperature and pH of the eluent were 60 °C and 7, respectively. When the concentration of TEA is increased, the retention of FLP increases. This is somewhat counterintuitive, as TEA causes a reduction in ON retention compared to AA. A possible explanation is that TEA suppresses electrostatic repulsion with unreacted silanols groups on the stationary phase, as also suggested by Wei et al. when AA is used in the eluent.^37^ Additionally, the presence of TEA in the mobile phase may decrease its overall hydrophilic character, further decreasing the retention.

**Figure 4:**
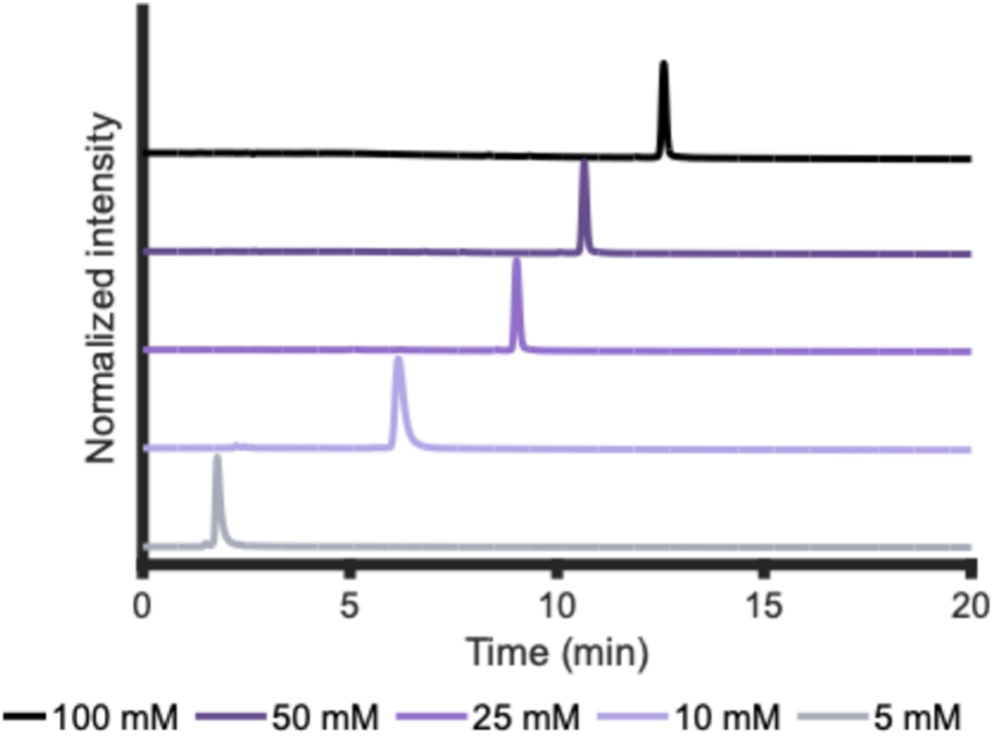
HILIC-UV chromatogram showing the effect of TEA concentration in the HILIC eluent (5, 10, 25, 50 mM) at pH 7 and column temperature of 60 °C for the analysis of the FLP. See the Experimental Section and Supporting Information S-V for other conditions and results.

Generally, increasing the concentration of TEA in the eluent reduced the observed peak widths (Figure S11) and had some effect on the separation selectivity. In particular, the 25 and 50 mM TEA concentrations presented the highest resolution between DA and FLP. Moreover, a higher TEA concentration resulted in a small improvement in separating the *N* – 1, *N* + 1, and PO impurities from the FLP. This minor improvement in resolution may be related to the decrease in the peak widths, but higher concentrations might also increase ON-IPR interactions. This results in more pronounced differences in ONs with varying numbers of nucleotides or ON-IPR interaction strengths (i.e., PO vs PS groups).

Finally, high concentrations of IPRs in the eluent cause ion suppression, detrimentally affecting MS detection. Therefore, we selected 25 mM as the optimal working condition.

### pH of the Eluent

The pH of the eluent affects the molecular charge of the ON, particularly when it is deaminated. For the tested FLP, deamination involves the conversion of 5-methylcytosine (MeC) to thymine (T), which goes along with the loss of an aromatic primary amine group (p*K*_a_ 4.6, basic) and a gain in acidity of the imide group (p*K*_a_ changes from 12.4 for MeC to 9.9 for T, acid). The difference in charge between the FLP and its deaminated form is a crucial parameter that contributes to their separation in WAX methods.^12^ Therefore, we studied the performance when using an eluent containing 25 mM TEA with a pH of 4.8, 7.0, and 9.0 (Figure S12; chromatograms in Figure S13).

An increase in the eluent pH leads to a decrease in retention of the ONs. This effect can be explained by the number of charges on the ON at different pH values. At pH 4.8, the amine group of the MeCs and adenines (A) (basic p*K*_a_ of 4.1) on the ON is partially positively charged, which decreases the net charge of the ON. However, the total number of charges on the ON is higher than at pH 7 (where all nucleobases are neutral), making the ON more hydrophilic and thus more strongly retained. At pH 9, T, G (p*K*_a_ 9.2, acid), and A (p*K*_a_ 9.8, acid) are partially negatively charged, increasing the number of IPR interaction sites. This increases the apparent hydrophobicity of ON and thus decreases its retention. Small variation of peak widths is observed at different pH conditions, with the smallest peak widths on average at pH 7 (Table S6).

When the pH is increased, the DA-FLP resolution improves slightly (Figure S12); however, for the other impurities, the separation with FLP worsens. The latter can be explained by considering the pH-induced changes in the charge of the ON species. An eluent pH of 6.3 was used in further experiments, as the separation is virtually identical to pH 7, but easier to prepare by adding equal molar concentrations of TEA and acetic acid.

### Column Temperature

Lardeux et al. showed that in HILIC, low temperatures are favorable for achieving diastereomer separation, while higher temperatures could disrupt ON conformations, leading to singular, sharp peaks.^33^ To assess the effect of column temperature on the separation of PS ONs in IP-HILIC, column temperatures of 5, 20, 45, 60, and 80 °C were tested (Figure S12; chromatograms in Figure S14). The TEA concentration and pH were kept at 25 mM and 7, respectively.

In our study, we observed that higher column temperatures lead to shorter ON retention times and smaller peak widths (Table S7). The latter can possibly be explained by decreased diastereomer separation at higher temperatures and/or an increase in mass transfer kinetics. The separation of the impurities from FLP also improved at higher temperatures. At 5 °C, the *N* – 1 and DA impurities coeluted with the FLP, while a resolution of approximately 1 was achieved for the *N* + 1 and PO impurity with respect to the FLP. In contrast, at 80 °C, resolutions of 1.5 or higher from the FLP were obtained for the *N* – 1, *N* + 1, DA, and PO impurities. Interestingly, the retention order of the *N* + 1 and PO impurities swapped when the temperature was above 45 °C, where the resolution between PO impurity vs FLP increased less than the *N* + 1 vs FLP when the temperature was raised. The steeper increase in resolution of *N* + 1 from FLP compared to that from PO might be related to the conformational differences of PO at low temperatures that are lost or reduced at high temperatures. The loss of conformations at higher temperatures might also impact the IPR-ON interactions as well as the interactions with the stationary phase, where at higher temperatures a larger surface area of the ON can interact due to unfolding. Moreover, for single guide RNA, it has been shown that peak shape remains constant in HILIC, while becoming sharper in both IP-RPLC and AEX when increasing the column temperature.^37–39^ Both IP-RPLC and AEX are dominated by electrostatic interactions for ON separations, while in HILIC, this contribution is minor. This suggests that electrostatic interactions play a more important role in IP-HILIC. Lastly, different temperature dependencies of the p*K*_a_ values of PO and PS might also play a role in the separation of the PO impurity from the FLP. However, the change in the DA-FLP selectivity is likely to be related to the increased hydrophilic interactions (relative) between the nucleobases and the stationary phase. A column temperature of 80 °C was chosen as optimal due to the overall highest separation resolution of each impurity from the FLP.

### IP-HILIC-MS for ON Impurity Profiling

For testing the potential of the optimized IP-HILIC method for impurity profiling, a mixture of FLP and *N* – 1, *N* + 1, PO, DA, and NC was prepared with the impurities at 2% in weight relative to FLP. The mixture was analyzed using IP-HILIC-UV-MS using an eluent comprising 25 mM TEAA (pH 6.3) and a column temperature of 80 °C. Figure 5 (top left) shows the obtained total-ion (TIC) and extracted-ion chromatograms (EICs) for each ON species. The TIC is dominated by the FLP signal, but in the EICs, the low-level impurities can be detected at suitable signal-to-noise ratios, confirming the good MS compatibility of the IP-HILIC method, despite the presence of TEAA as IPR. The detected impurities can now be selectively assigned according to their masses recorded with MS. The obtained impurity profile corresponds to what could be expected from the exploring IP-HILIC-UV experiments (Figure S14): the NC, *N* – 1, and DA impurities elute before the FLP, and the PO and *N* + 1 are eluting after FLP, indicating satisfactory LC-MS interfacing. The DA and *N* – 1 impurities are well separated from the FLP, coelute together. Nevertheless, as their masses differ significantly, they can still be adequately differentiated by MS.

**Figure 5:**
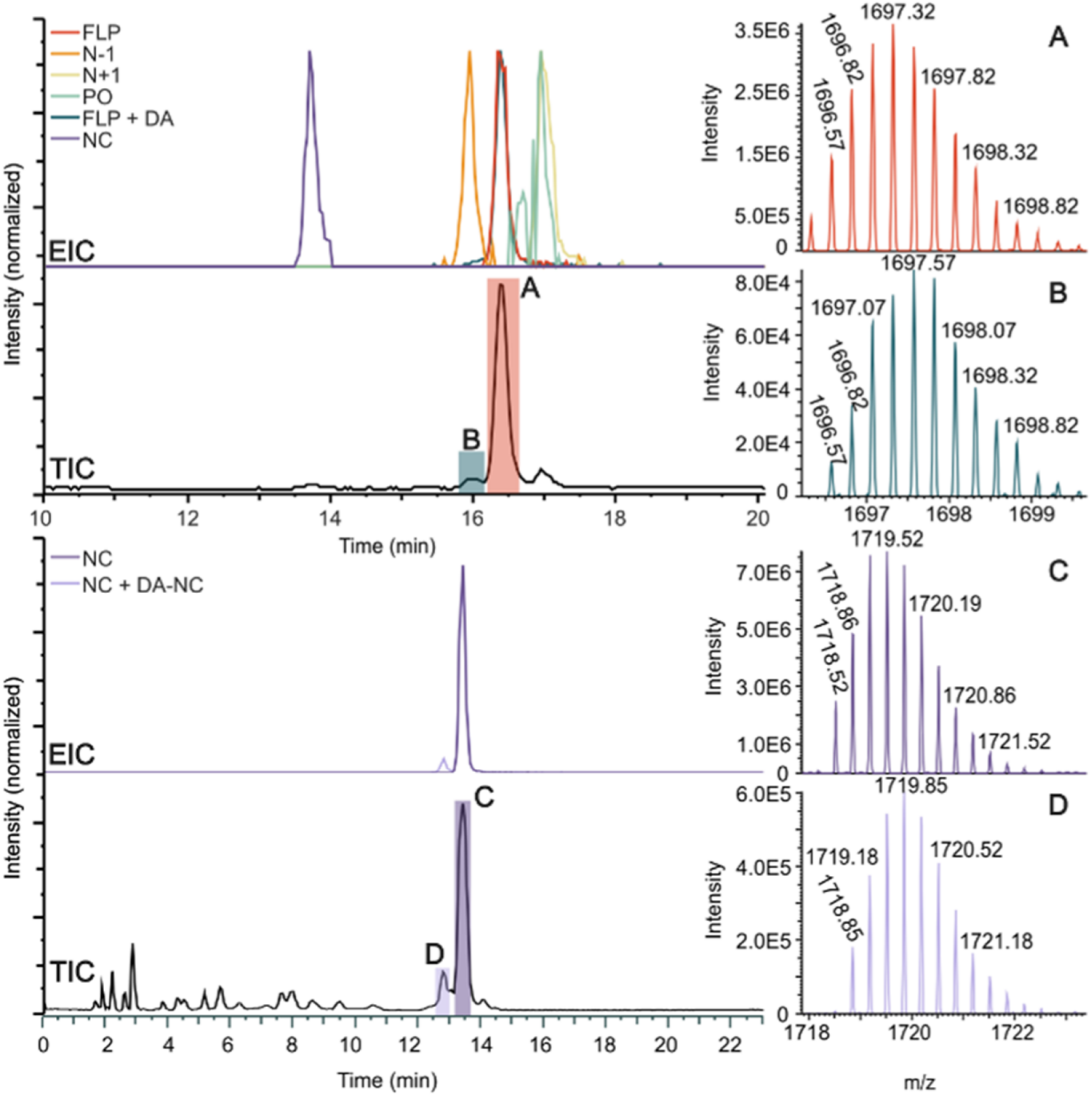
Left: IP-HILIC-UV-MS of (top) mixture of FLP and impurities at 2% and (bottom) mixture of NC with DA-NC at 2% total-ion chromatograms (TIC) and extracted-ion chromatograms (EICs; ±5 ppm *m*/*z*) for 1696.32 (FLP), 1611.07 (*N* – 1), 1783.58 (*N* + 1), 1694.33 (PO), 1697.32 (FLP and DA), 1719.52 (NC), and 1718.52 (NC and DA-NC) for the mixtures are plotted. Right: mass spectra showing the isotope patterns of the (A, B) [M – 4H]^4–^ ions and (C, D) [M – 3H]^3–^ ions observed in the peaks highlighted in the corresponding TICs. See the Experimental Section for the experimental conditions.

Notably, the separation of the DA impurity from the FLP is essential, as these differ by only less than 1 Da in mass, making their distinction based on MS only very challenging. The MS isotope patterns of DA and FLP strongly overlap, which hinders reliable assignment and quantification of DA in the presence of excess FLP, as would be the case for IP-RPLC, in which FLP and DA are hardly separated (Figures S7 and S8). Figure 5 (top right) provides the average mass spectra of the [M – 4H]^4–^ ion recorded for the peaks at the retention times of FLP and DA (indicated as A and B, respectively). A clear shift in the isotopic pattern is observed, with the pattern of peak B being shifted one mass unit higher. This shift confirms the presence of the DA impurity in the highlighted B section. Further confirmation was obtained by charge deconvolution of the full mass spectra (Figures S15 and S16), which resulted in 6793 and 6794 Da for peaks A and B, respectively (i.e., the molecular masses of the FLP and DA impurity, respectively). To further showcase the obtained separation of DA from the FLP, EICs for the most abundant isotope of FLP (*m*/*z* 1697.32) and the first isotope observed for FLP (*m*/*z* 1696.32) was plotted (Figure 5, red and teal traces, respectively). The most abundant isotope observed for FLP coincides with the second most abundant isotope observed for DA, and therefore, both FLP and DA are visible in the EIC of *m*/*z* 1697.32. The first isotope of FLP with *m*/*z* 1696.32 is lacking in the mass spectrum of the DA impurity. Indeed, by comparing the two constructed EICs, a small peak preceding the FLP peak can be observed in the *m*/*z*-1697.32 EIC, while this peak is not present in the *m*/*z*-1696.32 EIC. This again indicates that the small preceding peak corresponds to the DA impurity.

The selectivity of IP-HILIC was further tested by the analysis of a mixture of NC and DA-NC with the DA-NC impurity at 2% weight relative to that of NC. Figure 5 (bottom left) shows the obtained TIC and EICs for the ON species, and Figure 5 (bottom right) provides the average mass spectra of the [M – 3H]^3–^ ion recorded for the peaks at the retention times of NC and DA-NC (indicated as C and D, respectively). Deconvolution of the recorded mass spectra (Figures S20 and S21) yielded masses of 5162 and 5163 Da for peaks C and D, respectively, indicating the small peak preceding the large NC peak corresponds to the DA-NC impurity. The observed shift of the isotope pattern (Figure 5, bottom right) and the construction of the DA-NC, revealing an EIC of *m*/*z* 1718.52 (analogous to the conjugated derivative above), confirmed the DA-NC assignment. These examples clearly demonstrate the potential of IP-HILIC-MS for reliable assignment of deaminated products in phosphorothioated ON pharmaceuticals.

In order to evaluate the separation space provided by IP-HILIC and probe its potential for broad impurity profiling, we analyzed the NC sample spiked with DA-NC by both IP-HILIC-MS and IP-RPLC-MS. The NC is a product purified to a lesser degree and contains various impurities next to non-GalNAc-conjugated FLP. Figure S19 shows the obtained TICs together with 19 EICs of the [M – 2H]^2–^ or [M – 2H]^3–^ ions of different components that could be discerned in the sample. For IP-HILIC-MS, one peak eluted after the NC peak at 14.1 min, showing a deconvoluted mass of 5145.62 Da. The difference of 16 Da with the NC (5162.58 Da) suggests the peak at 14.1 min corresponds to a PO impurity. Interestingly, as for the DA-NC, this PO impurity was not separated from the NC peak by IP-RPLC-MS (Figure S19). Most peaks observed had shorter retention times than NC and all revealed masses smaller than the NC, differing 300 Da (i.e., the approximate mass of one nucleotide) or more. These most probably are shortmer impurities, which indeed exhibit shorter retention in both IP-HILIC and IP-RPLC. For IP-HILIC, peaks at 13.0 and 12.6 min could be assigned tentatively to *N* – 1 (4832.56 Da; loss of dG) and N-2 (4503.53 Da; loss of 2 dG) impurities, respectively. Tentative assignment of the other observed peaks was not attempted; most likely, the underlying species result from two or more failures and/or degradations. Overall, Figure S19 shows that retention in both IP-HILIC and IP-RPLC increases with ON size, but some specific differences in separation selectivity can be observed. Most essential difference, as indicated above for the DA and PO impurities, is that IP-HILIC is capable of separating impurities that comprise the same number of nucleotides. At the same time, IP-HILIC provides a satisfactory separation of the other components, facilitating impurity assignment by MS.

## Conclusions

This study focused on the use of IP-HILIC-MS for the separation and assignment of impurities of phosphorothioated ONs comprising the same number of nucleotides as the FLP. We systematically studied the influence of IP-HILIC separation parameters, such as type and concentration of IPR, eluent pH, and column temperature, using a poly­(dT) ladder and a model FLP (GalNAc-conjugated PS 16-mer ASO) with several of its related impurities (*N* – 1, *N* + 1, PO, DA, and NC). We showed that IPRs in the HILIC eluent (i) reduce the relative contribution of the highly polar phosphate moieties to ON retention, (ii) alter the HILIC separation selectivity inducing resolution of DA, PO, and NC impurities from the FLP while maintaining separation of *N* – 1 and *N* + 1 from the FLP, and (iii) reduce peak widths of phosphorothioated ONs, most probably due to reduction of diastereomer separation. Optimal resolution for the model FLP and its related impurities was obtained using an IP-HILIC eluent of pH 6.3 with 25 mM TEA, and a column temperature of 80 °C. The feasibility of the optimized method for impurity profiling was indicated by IP-HILIC-MS analysis of the model FLP containing several impurities at the 2% level. Comparison with current IP-RPLC, AEX, and HILIC methods showed that only IP-HILIC was able to resolve the DA product, an essential but difficult to detect impurity, from the FLP. Moreover, the selectivity and sensitivity IP-HILIC-MS showed the suitability of revealing a large number of minor impurities in an unpurified standard ON. Therefore, we believe that IP-HILIC-MS shows interesting potential for impurity profiling of therapeutic ONs.

## Supplementary Material



## Notes

IP-HILIC retention mechanism, all IP-HILIC chromatograms, peak width summaries, IP-RPLC and AEX chromatograms, and LC-MS data with mass deconvolution (PDF)
