# Supplementary material for: Ion-Pairing Hydrophilic Interaction Chromatography for Impurity Profiling of Therapeutic Phosphorothioated Oligonucleotides
Source: Anal Chem. 2025 Jul 17;97(29):15717–26. doi: 10.1021/acs.analchem.5c01407 (PMC12311887; doi:10.1021/acs.analchem.5c01407)
Supplement: Supplementary file 1 [file ac5c01407_si_001.pdf]

Supporting Information

Ion-pairing hydrophilic interaction chromatography for impurity profiling of therapeutic phosphorothioated oligonucleotides

Luca Tutiš[1,2]*, Paul D. Ferguson[3], David Benstead[4], Adrian Clarke[5], Carl Heatherington[6], Chris Gripton[6], Christina J. Vanhinsbergh[3], Govert W. Somsen[1,2], Andrea F. Gargano[2,7]*

[1] Division of BioAnalytical Chemistry, Department of Chemistry and Pharmaceutical Sciences, Amsterdam Institute of Molecular and Life Sciences (AIMMS), Vrije Universiteit Amsterdam, de Boelelaan 1085, 1081 HV Amsterdam, the Netherlands

[2] Centre for Analytical Sciences Amsterdam, Science Park 904, 1098 XH Amsterdam, The Netherlands

[3] New Modalities and Parenteral Development, Pharmaceutical Technology & Development, AstraZeneca Macclesfield, Charter Way SK10 2NA, UK

[4] Chemical Development, Pharmaceutical Technology & Development, AstraZeneca, Macclesfield, Charter Way SK10 2NA, UK

[5] Novartis Pharma AG, Fabrikstrasse 2, CH-4056 Basel, Switzerland

[6] GlaxoSmithKline, Gunnels Wood Rd SG1 2NY, Stevenage, UK

[7] Van 't Hoff Institute for Molecular Sciences, University of Amsterdam, Science Park 904, 1098 XH Amsterdam, The Netherlands

Table of content

S-I – Oligonucleotide sequence and modifications 2

S-II – Schematic representation of IP-HILIC retention of ONs 3

S-III – Effect of IPR type on IP-HILIC impurity separation 4

S-IV –IP-RPLC and AEX of model FLP and related impurities 8

S-V – IP-HILIC: effect of IPR concentration, eluent pH, and column temperature 11

S-VI – IP-HILIC-MS of model FLP and related impurities 14

S-I – Oligonucleotide sequence and modifications

The full sequence and modifications of the ONs used in this study are shown in Table S1 along with the average molecular mass. The GalNAc-conjugated ONs comprise of 2-deoxyribose sugars, thioated phosphate groups, and guanine (G), 5-methylcytosine (MeC), adenine (A), thymine (T). The 8-mer comprises of 2' fluoro (f), 2'-O-methylation (o), and 2-deoxyribose sugars, 2 thioated phosphate groups, and cytosine (C), uracil (U), G, A, and T.

Table S1: Model PS ON names and sequences (incl. modifications) from 5'-3' used in this study.

Name	Sequence[1]	Avg. molecular mass (Da)
FLP	GalNAc dG dMeC dA dT dT dMeC dT dA dA dT dA dG dMeC dA dG dMeC	6793.98
DA	GalNAc dG dT dA dT dT dMeC dT dA dA dT dA dG dMeC dA dG dMeC	6794.96
PO	GalNAc dG dMeC dA dT dT dMeC dT dA *dA dT dA dG dMeC dA dG dMeC	6777.92
N+1	GalNAc dG dG dMeC dA dT dT dMeC dT dA dA dT dA dG dMeC dA dG dMeC	7139.25
N-1	GalNAc dMeC dA dT dT dMeC dT dA dA dT dA dG dMeC dA dG dMeC	6448.71
Switch (dT/dG)	GalNAc dT dMeC dA dG dG dMeC dG dA dA dG dA dT dMeC dA dT dMeC	6819.00
NC	dG dMeC dA dT dT dMeC dT dA dA dT dA dG dMeC dA dG dMeC	5162.25
DA-NC	dG dT dA dT dT dMeC dT dA dA dT dA dG dMeC dA dG dMeC	5163.23
8-mer	oC fA oA *oG *oU *fC *fG *dT	2601.80

[1]PO linkage (*)

S-II – Schematic representation of IP-HILIC retention of ONs

In RPLC, the ON is poorly retained on the stationary phase in the absence of IPRs due to the hydrophilic nature of the ON. When positively charged IPRs are added to the eluent, the negative charges on the backbone are neutralized and the apparent hydrophobicity is increased, increasing the retention on the hydrophobic stationary phase. Therefore, a separation based on the number of phosphate groups (i.e., nucleotides) can be obtained. This is shown in Fig. S1.

In HILIC, the highly polar phosphate moieties contribute significantly to the retention of ONs, masking the slight differences in hydrophilicity on the other parts of the ONs, such as the deamination of 5-methylcytosine to thymine. By adding positively charged IPRs to the eluent, the apparent hydrophilicity of the phosphate moieities is reduced. Thereby, the hydrophilicity of the nucleobases and GalNAc-conjugate group is indirectly increased in relative terms. This increases the relative contribution of the nucleobases and GalNAc group on retention, whilst decreasing the relative contribution of the phosphate groups. This effect is visualized on Fig. S1.

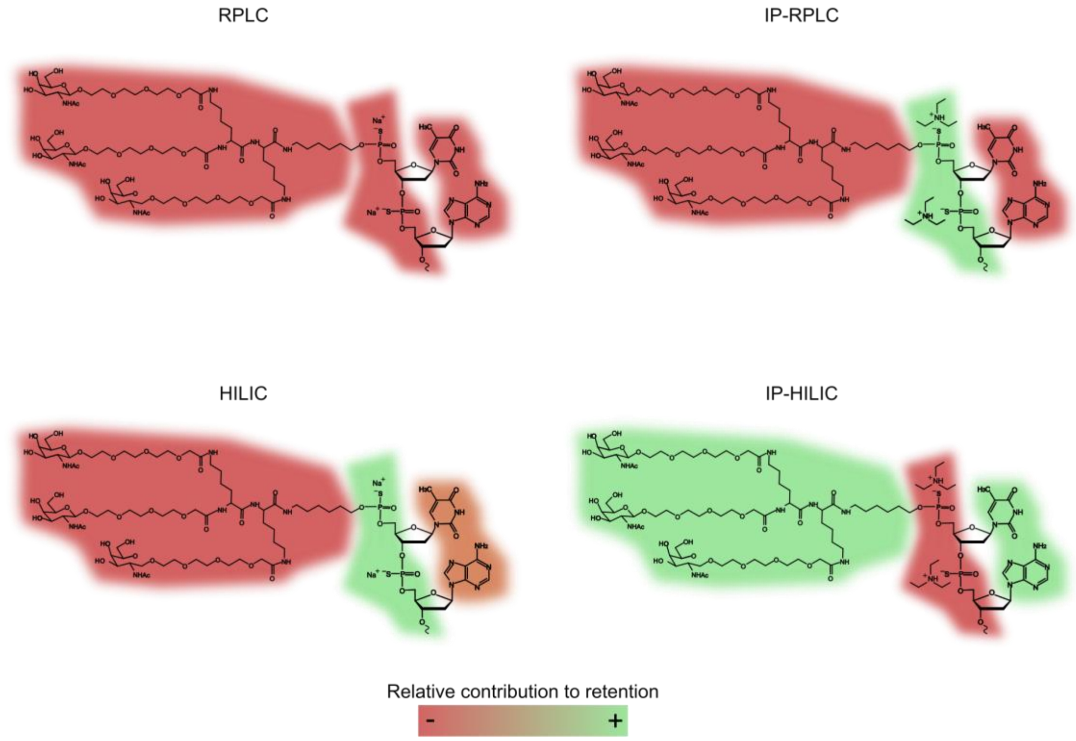

Figure S1: Schematic representation of the proposed effect of IPRs in the HILIC eluent on the retention of ONs

S-III – Effect of IPR type on IP-HILIC impurity separation

Baseline drifts were observed when IPRs were added to the HILIC eluent. As these drifts were relatively large in comparison to the intensity of the ONs in the poly(dT) ladder, baseline subtractions were performed by subtracting system blank measurements (Fig. S2).

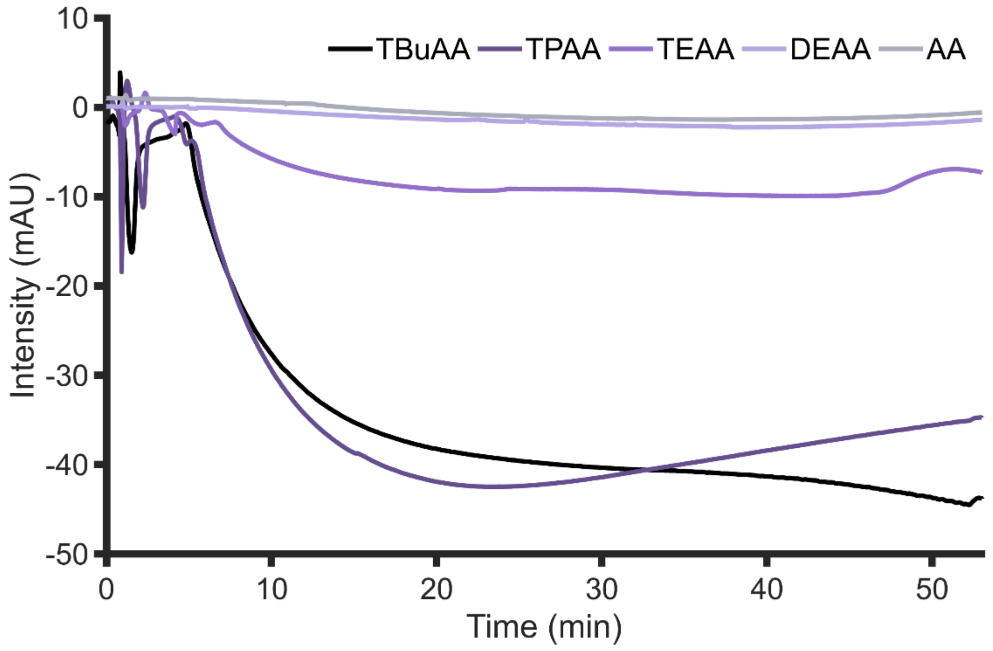

Figure S2: Baselines observed during IP-HILIC-UV of system blanks using an eluent containing 15 mM of AA, DEAA, TEAA, TPAA, or TBuAA (pH 7) and a column temperature of 45 °C. Other conditions, see Experimental Section.

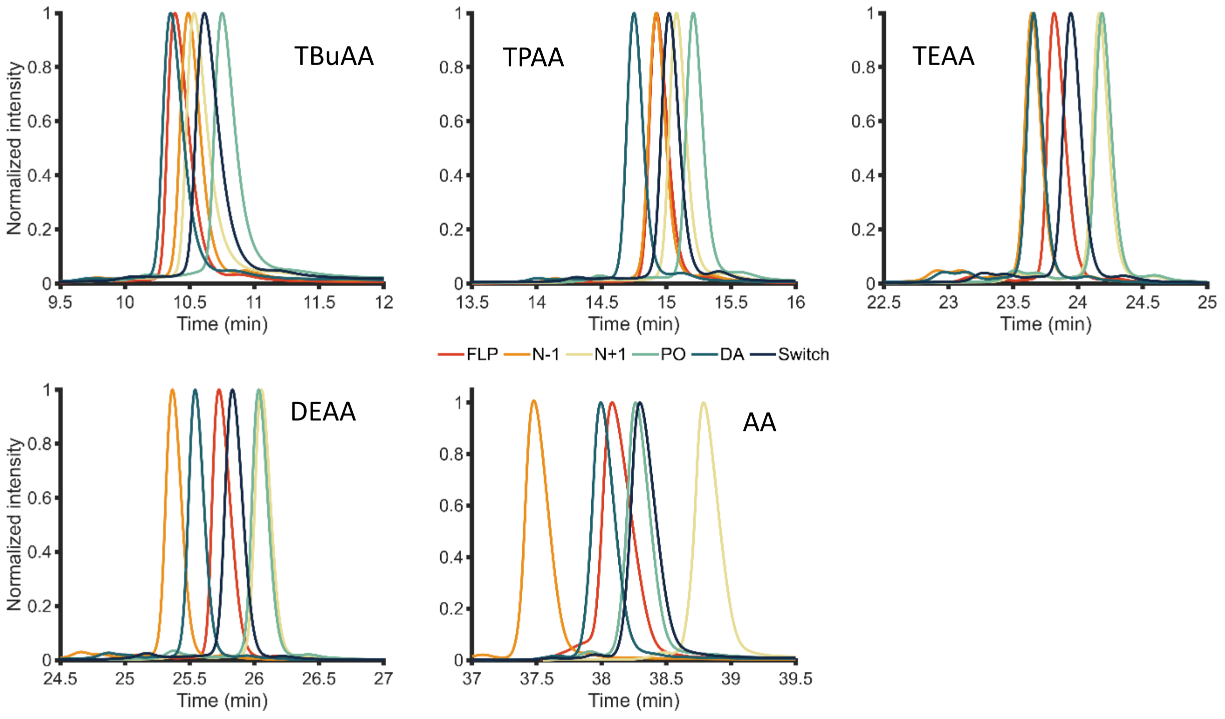

Figure S3: HILIC-UV of the model FLP and related impurities using the same conditions as indicated for Fig. S2.

From Fig. 1 and Fig. S3 and S4, peak widths at half-height were extracted and noted in Table S1 and S2, respectively. For the poly(dT) ladder, peak capacities are also indicated and calculated using Eq. 1.

Table S2: Peak widths at half-height (in min) and peak capacities observed during IP-HILIC of the poly(dT) ladder ONs using an eluent with 15 mM AA or indicated IPR; data taken from chromatograms shown in Fig. 1. Other conditions, see Materials and Methods.

Poly(dT)/IPR	AA	DEAA	TEAA	TPAA	TBuAA
15-mer	0.106	0.110	0.126	0.147	0.283
20-mer	0.109	0.112	0.128	0.166	ND
25-mer	0.117	0.124	0.148	0.213	ND
30-mer	0.129	0.138	0.177	0.288	ND
35-mer	0.141	0.155	0.193	0.347	ND
n_c	258	243	201	134	ND
n_c^{eff}	35	22	14	7	ND

ND, not determinable

Table S3: Peak widths at half-height (in min) observed during IP-HILIC of the FLP and related impurities using an eluent with 15 mM AA or indicated IPR; data taken from chromatograms shown in Figs. S3. Other conditions, see Experimental Section.

ON/IPR	AA	DEAA	TEAA	TPAA	TBuAA
FLP	0.248	0.159	0.153	0.157	0.185
N-1	0.200	0.140	0.140	0.147	0.173
N+1	0.205	0.138	0.139	0.146	0.183
PO	0.198	0.140	0.142	0.147	0.181
DA	0.195	0.135	0.137	0.142	0.176
Switch	0.214	0.155	0.157	0.155	0.197

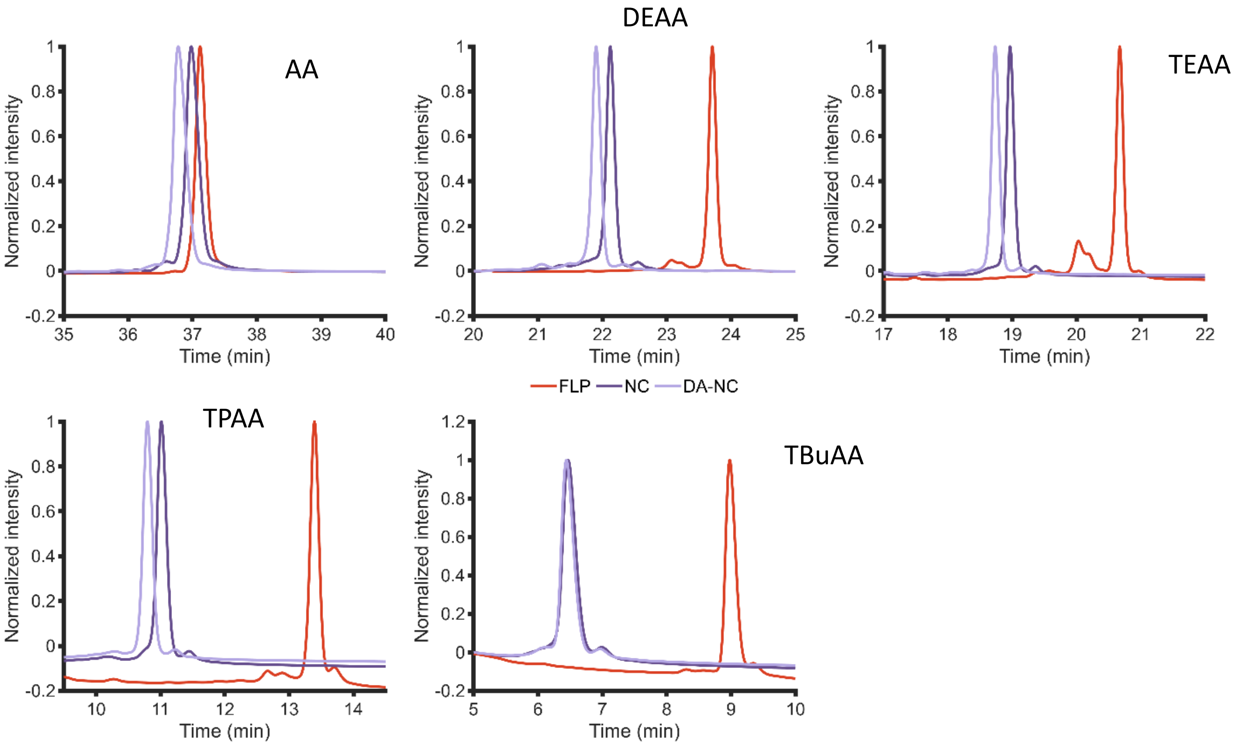

Figure S4: HILIC-UV of FLP, NC and DA-NC using the same experimental conditions as described for Fig. S2.

Table S4: Peak widths at half-height (in min) observed during IP-HILIC of NC and DA-NC using an eluent containing 15 mM AA or indicated IPR; data taken from chromatograms shown in Fig. S4. Other conditions, see Experimental Section.

ON/IPR	AA	DEAA	TEAA	TPAA	TBuAA
NC	0.22	0.148	0.134	0.165	0.232
DA NC	0.223	0.152	0.136	0.161	0.213

The peak widths at half height noted in Table S1 and S2 were also plotted in Fig. S5. Here, you can clearly see that the PS ONs have a deviating trend compared to the 15-mer of the poly(dT) ladder.

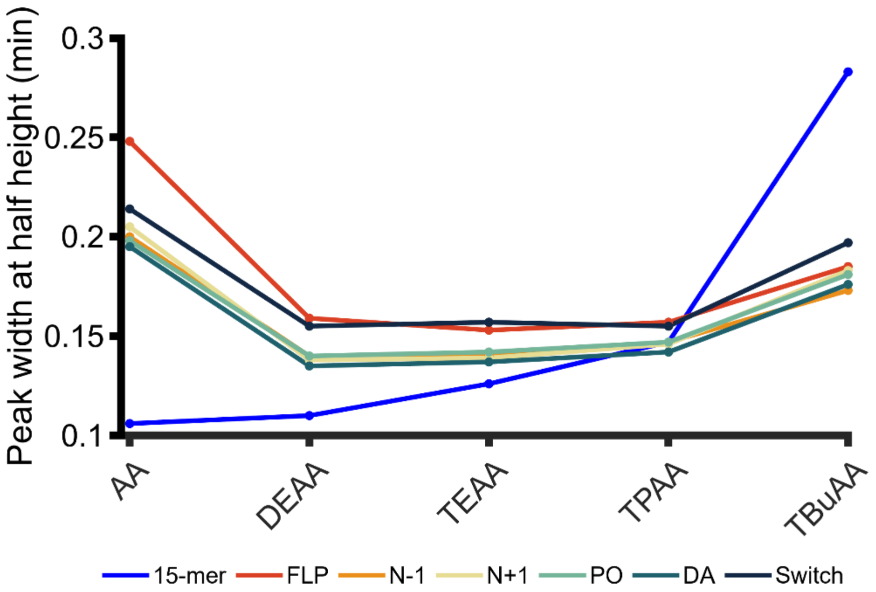

Figure S5: Peak widths at half height (min) of the PS ONs and the 15-mer of the poly(dT) ladder in the presence of various IPRs in the eluent using the same experimental conditions as indicated for Fig. S2.

To highlight the changes in the degree of diastereomer separation when IPRs are added to the HILIC eluent, an 8-mer comprising of 2 PS modifications (4 diastereomers) was tested using the same experimental conditions as for the poly(dT) ladder and GalNAc-conjugated PS ONs (Fig. S5). Here, a clear reduction in diastereomer separation can be observed when the IPR hydrophobicity is increased. When AA is used in the eluent, a partial separation of the 4 diastereomers can be observed, whilst for DEAA and TEAA, a singular peak can be observed with a slight shoulder appearing on the right side of the peak. For TPAA and TBuAA, only a singular peak was observed.

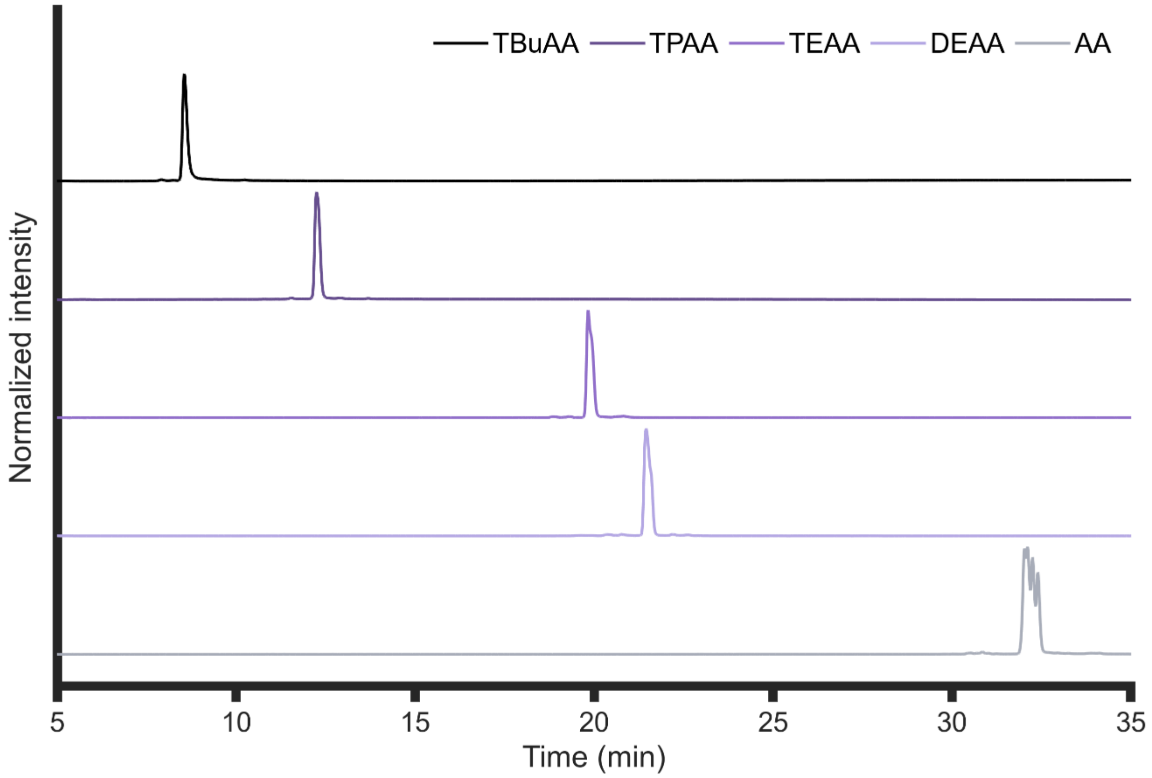

Figure S6: HILIC-UV of the 8-mer ON using the same experimental conditions as indicated for Fig. S2.

S-IV –IP-RPLC and AEX of model FLP and related impurities

To showcase the differences in selectivity between IP-HILIC method and IP-RPLC and AEX using standard conditions, the model FLP and the related impurities were also measured using these LC modes. In Fig. S6 and S7, the chromatograms of the IP-RPLC method are shown. A clear separation of N-1 and N+1 from the FLP can be observed, whilst the remaining impurities elute with a similar retention time as the FLP.

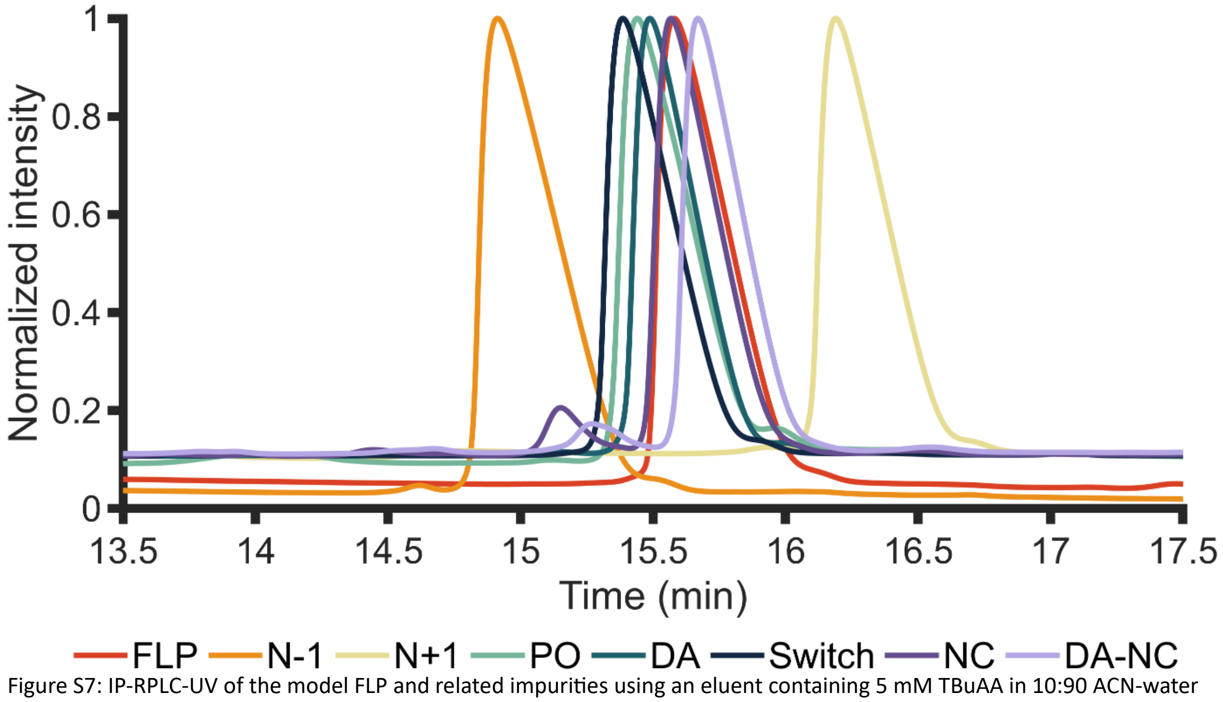

Figure S7: IP-RPLC-UV of the model FLP and related impurities using an eluent containing 5 mM TBuAA in 10:90 ACN-water (v/v) (A), and 5 mM TBuAA in 80:20 ACN-water (v/v) (B) on a BEH C18 column 60 °C. Other conditions, see Experimental Section.

Due to many peaks eluting close to the FLP, the FLP-DA and NC-DA-NC separations are plotted in Fig. S7. Clearly, the deamination impurities are not resolved from the FLP or NC.

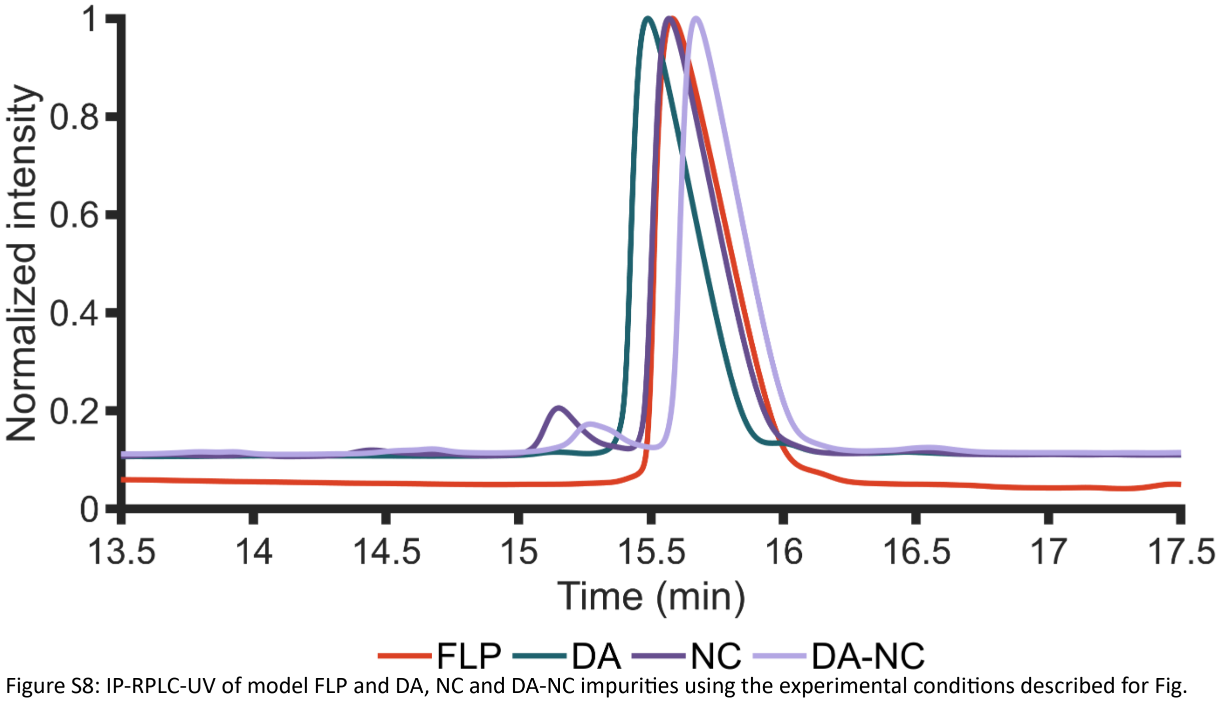

Figure S8: IP-RPLC-UV of model FLP and DA, NC and DA-NC impurities using the experimental conditions described for Fig. S6.

In Fig. S8 and S9, the chromatograms of the AEX method are shown. Similarly, a separation of N-1 and N+1 from the FLP can be obtained. The oxidation of the phosphorothioate to the phosphorothioate also results in a difference in acidity, where PS has a lower pK_a (more acidic). Therefore, the PO impurity eluted before the FLP, whilst co-elution of this impurity was observed with IP-RPLC. Moreover, the NC and DA-NC are resolved well from the FLP and have a larger retention time compared to the FLP. This may suggest that the GalNAc group causes steric hindrance to the phosphate backbone.

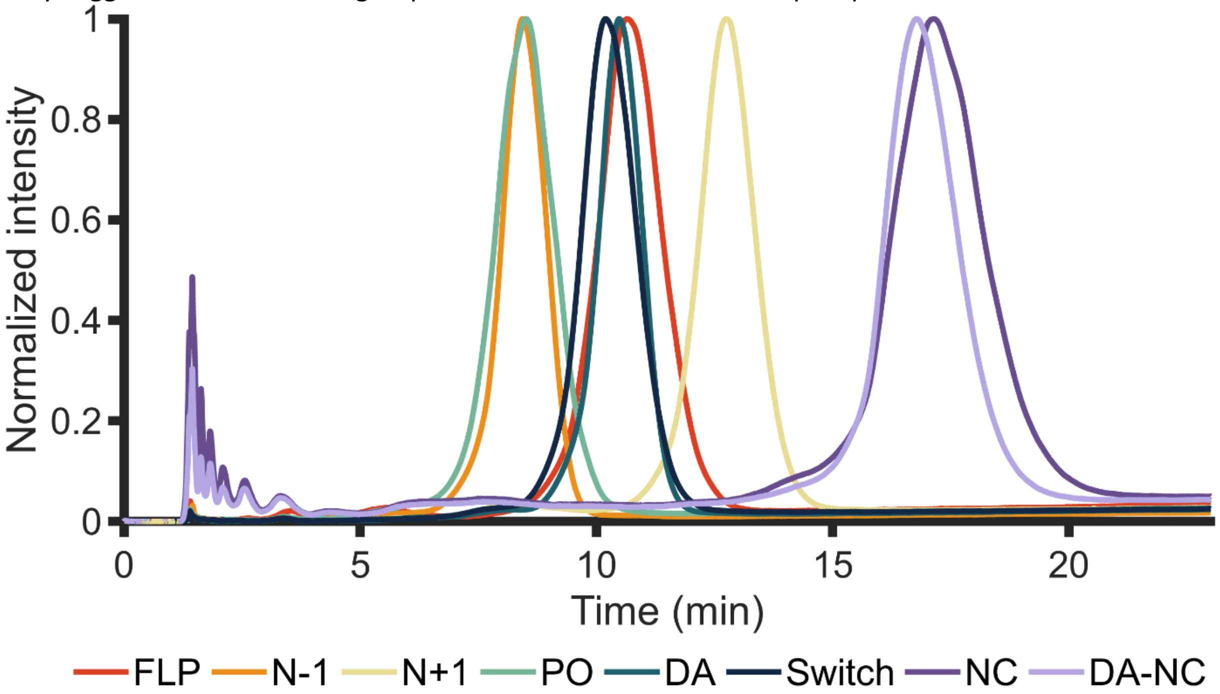

Figure S9: AEX-UV of model FLP and related impurities using an eluent containing 20 mM Tris-HCl in 10:90 ACN-water (v/v) (A) and 2M NaCl in 10:90 ACN-water (v/v) (B) on a TOSOH TSKgel DNA-STAT column at 25 °C. Other conditions, see Experimental Section.

Also here, the FLP-DA and NC-DA-NC separations are plotted in a separate chromatogram to highlight that the deamination impurities are not resolved from their non-deaminated counter parts.

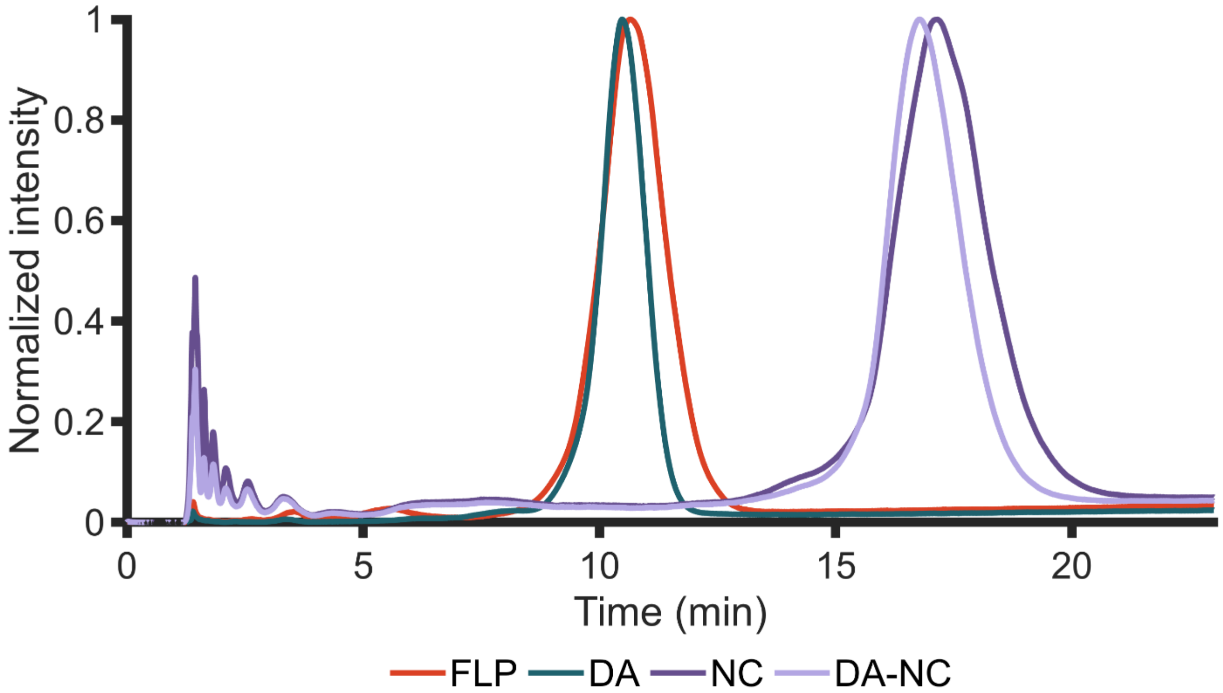

Figure S10: AEX-UV of model FLP and DA, NC and DA-NC impurities using the experimental conditions described for Fig. S8.

S-V – IP-HILIC: effect of IPR concentration, eluent pH, and column temperature

To further improve the separation of deamination impurities from the FLP, the TEA concentration in the eluent was varied between 5 and 100 mM, and the effect on peak width, MS intensity, and resolution between species was studied.

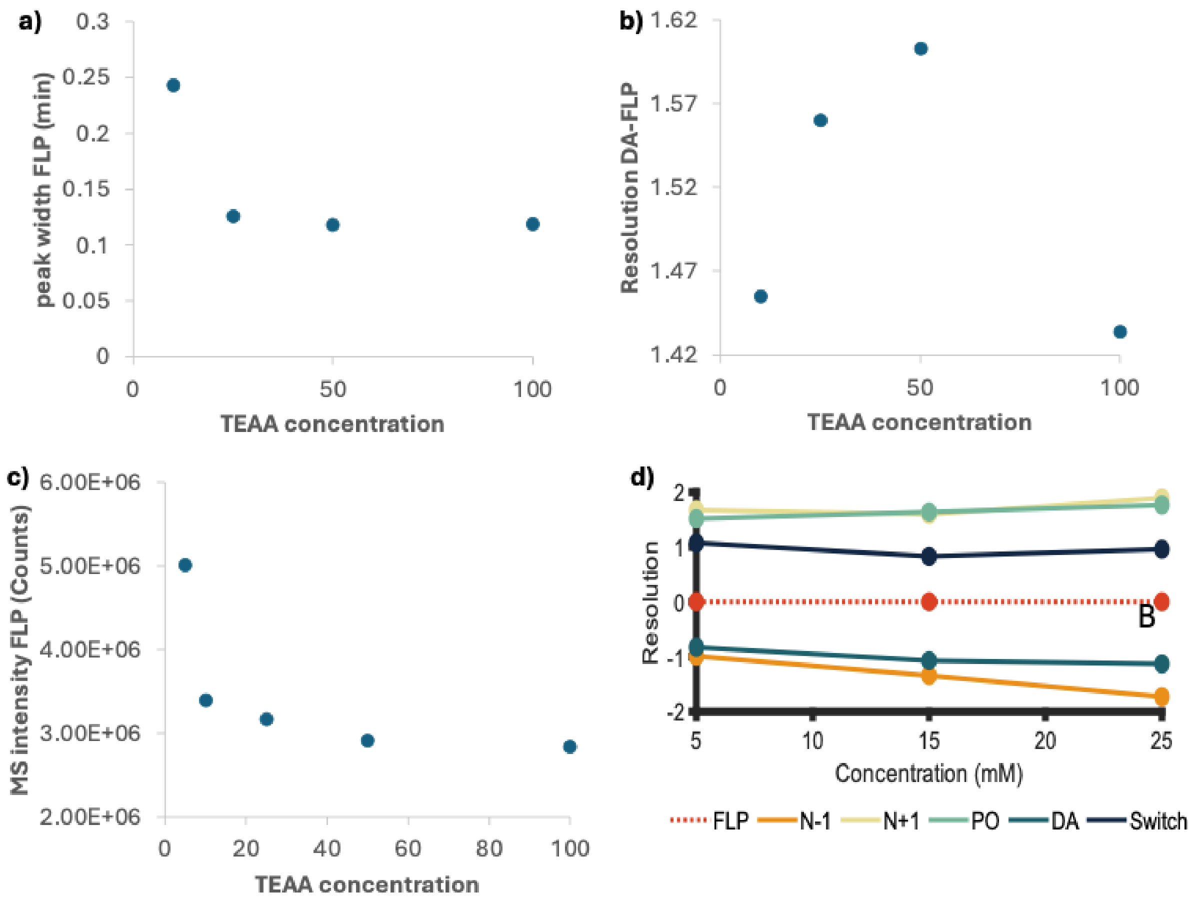

Figure S11: The effect of TEA concentration in the HILIC eluent at pH 7 and column temperature of 60 °C on the separation of the model FLP and its related impurities. Other conditions, see Materials and Methods except for results from d) that are obtained using a Acquity UPLC BEH amide column (2.1 x 150 mm, 1.7 µm dp, 130 Å), eluent A consisted of milli-Q water with TEAA and eluent B of ACN with TEAA. The gradient started with a 1-min hold at 80% B, followed by a linear decrease to 66.7% B from 1-21 min, and a hold at 66.7% B for 2 min. The flow rate was 0.2 mL/min.

Similarly, the eluent pH and column temperature were optimized to improve the separation of deamination as well as the other impurities from the FLP. The eluent pH was varied between 4.8 to 9 and the column temperature between 5 to 80 °C. The effect of these parameters on the retention of the FLP and the resolution of the impurities with the FLP are shown in Fig. S11.

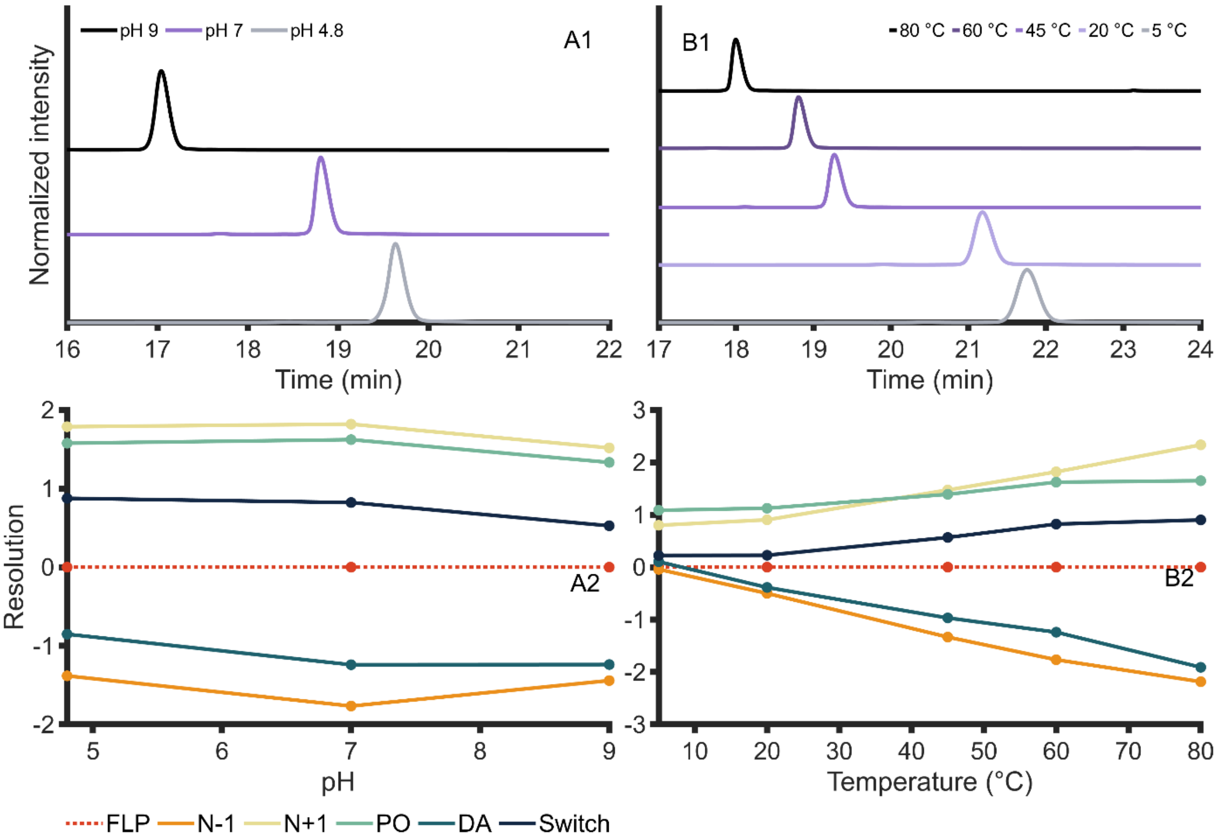

Figure S12: The effect of (A) eluent pH (4.8, 7.0, 9.0) and (B) column temperature (5, 20, 45, 60, 80 °C) on the retention (A1 and B1) and the resolution (A2 and B2) of the model FLP and its impurities. Other conditions, see Experimental Section.

The chromatograms of the model FLP and its related impurities at various eluent pH values are shown in Fig. S12. From these chromatograms, peak widths at half-height were extracted and noted in Table S5.

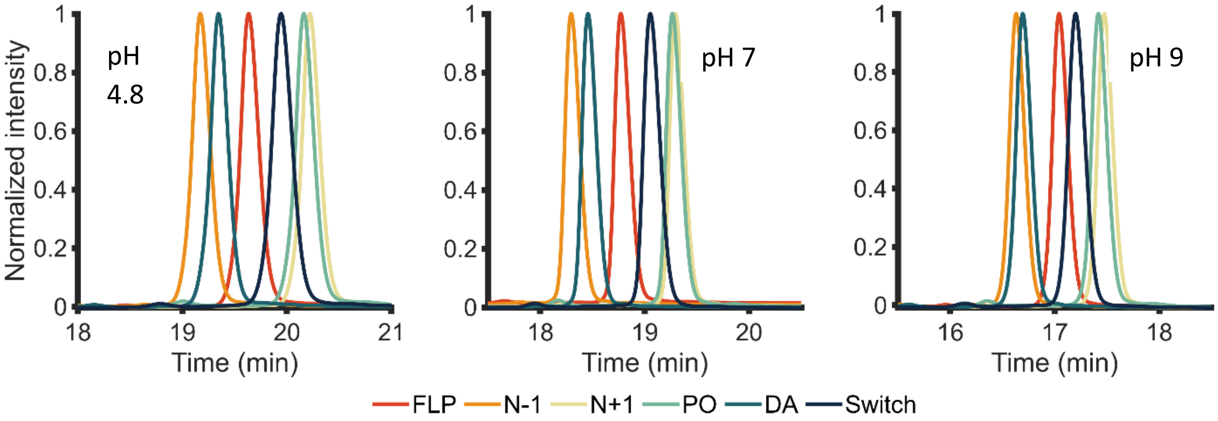

Figure S13: The effect the eluent pH (4.8, 7.0, 9.0) at 25 mM TEAA and a column temperature of 60 °C on the separation of the model FLP and its related impurities. Other conditions, see Experimental Section.

Table S6: Peak widths at half-height (in min) observed during IP-HIILC of the model FLP and its related impurities using an eluent containing the indicated eluent pH; data taken from chromatograms shown in Fig. S12. Other conditions, see Experimental Section.

ON/eluent pH	pH 9	pH 4.8	pH 7
FLP	0.168	0.197	0.164
N-1	0.166	0.194	0.162
N+1	0.165	0.191	0.159
PO	0.167	0.198	0.162
DA	0.164	0.189	0.158
Switch	0.189	0.219	0.179

The chromatograms of the model FLP and its related impurities at various eluent pH values are shown in Fig. S13. Peak widths at half-height were extracted from these chromatograms and noted in Table. S6.

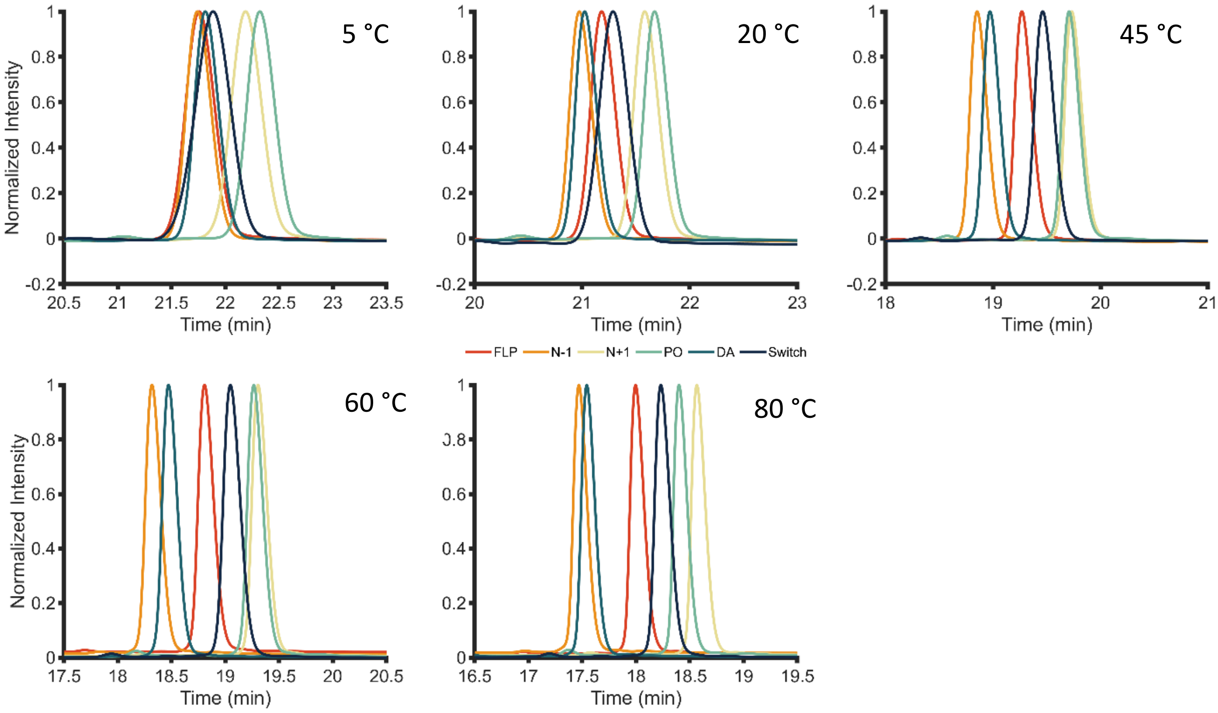

Figure S14: The effect of column temperature (5, 20, 45, 60, 80 °C) at 25 mM TEAA and pH 7 on the separation of the model FLP and its related impurities. Other conditions, see Experimental Section.

Table S7: Peak widths at half-height (in min) of the model FLP and its related impurities using an eluent with the indicated pH; data taken from chromatograms shown in Fig. S13.

ON/Temp	5 °C	20 °C	45 °C	60 °C	80 °C
FLP	0.308	0.26	0.187	0.164	0.143
N-1	0.263	0.234	0.183	0.162	0.142
N+1	0.324	0.260	0.180	0.159	0.144
PO	0.298	0.252	0.185	0.162	0.142
DA	0.253	0.226	0.177	0.158	0.140
Switch	0.375	0.308	0.207	0.179	0.157

S-VI – IP-HILIC-MS of model FLP and related impurities

Deconvolution of mass spectra obtained of ONs with IP-HILIC was performed using Unidec applying the following settings: charge range, 2 – 5; mass range, 4000 – 8000; Sample mass, every 1 Da; negative mode; peak detection range, 1 Da; peak detection threshold, 0.1. Mass spectra of ONs obtained during IP-HILIC-MS under optimized conditions: Fig. S14, FLP (6793 Da); Fig. S15, N-1 (6449 Da) and DA (6794 Da); Fig. S16, N+1 (7138 Da) and PO (6777 Da); Fig. S17 NC (5162 Da). The additionally detected masses listed in the figures originate from ON adducts.

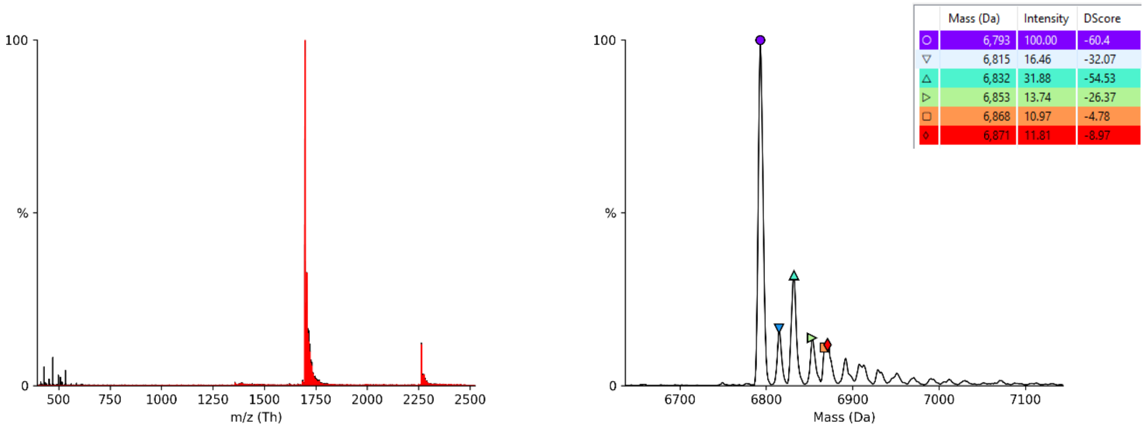

Figure S15: Mass spectrum and deconvoluted mass spectrum of peak eluting at 16.20 min during IP-HILIC-MS of test mixture (see Fig. 4 top). Red indicated m/z window is used for deconvolution.

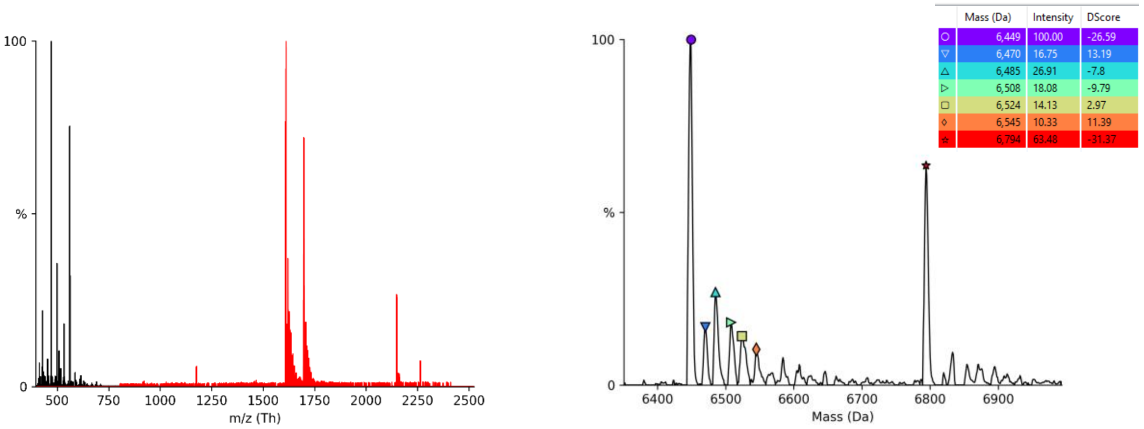

Figure S16: Mass spectrum and deconvoluted mass spectrum of peak eluting at 15.94 min during IP-HILIC-MS of test mixture (see Fig. 4 top). Red indicated m/z window is used for deconvolution.

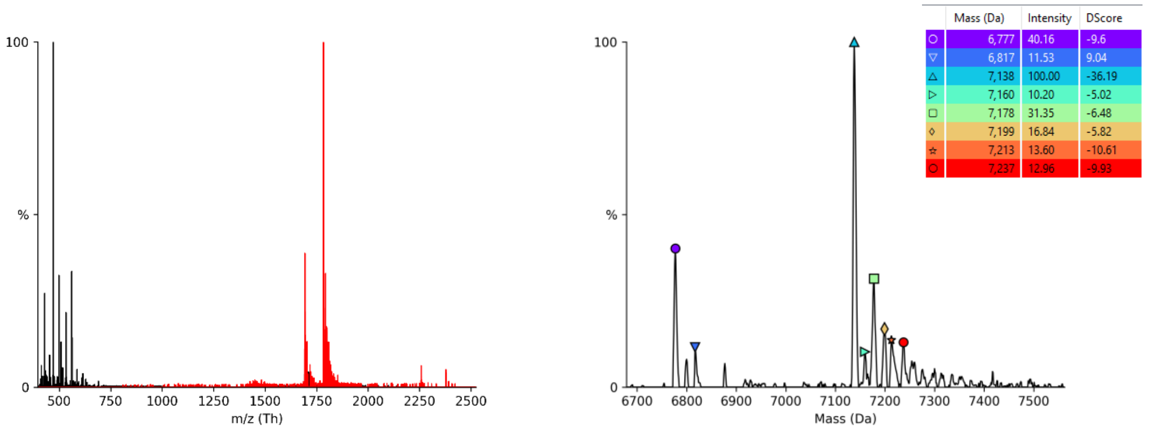

Figure S17: Mass spectrum and deconvoluted mass spectrum of peak eluting at 16.98 min during IP-HILIC-MS of test mixture (see Fig. 4 top). Red indicated m/z window is used for deconvolution.

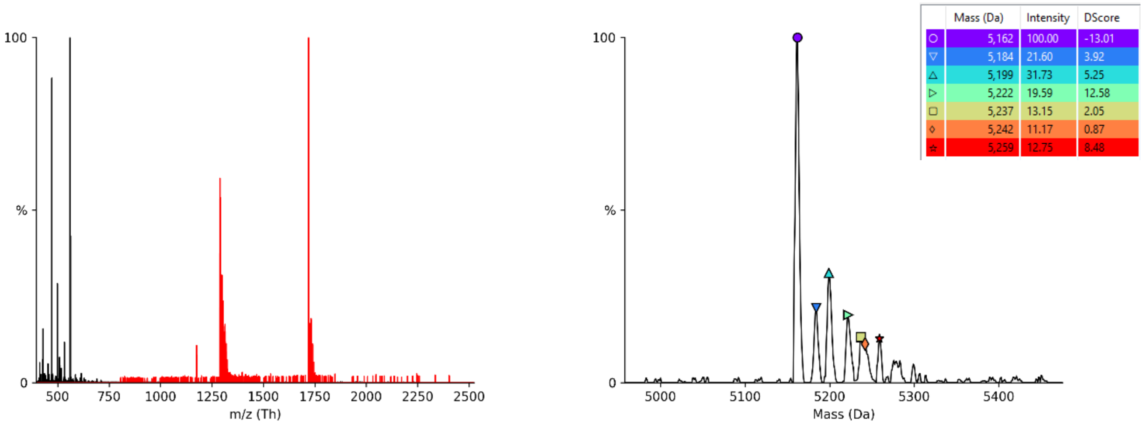

Figure S18: Mass spectrum and deconvoluted mass spectrum of peak eluting at 13.72min during IP-HILIC-MS of test mixture (see Fig. 4 top). Red indicated m/z window is used for deconvolution.

Fig. S18 shows the TIC and EICs obtained for IP-HILIC-MS and IP-RPLC-MS of the NC and DA-AC mixture. Next to NC and DA-NC a large number of unknown synthesis impurities were detected originating from the unpurified NC sample used to make the mixture. A few peaks have been tentatively assigned based on recorded mass (see last paragraph of Section 3.3).

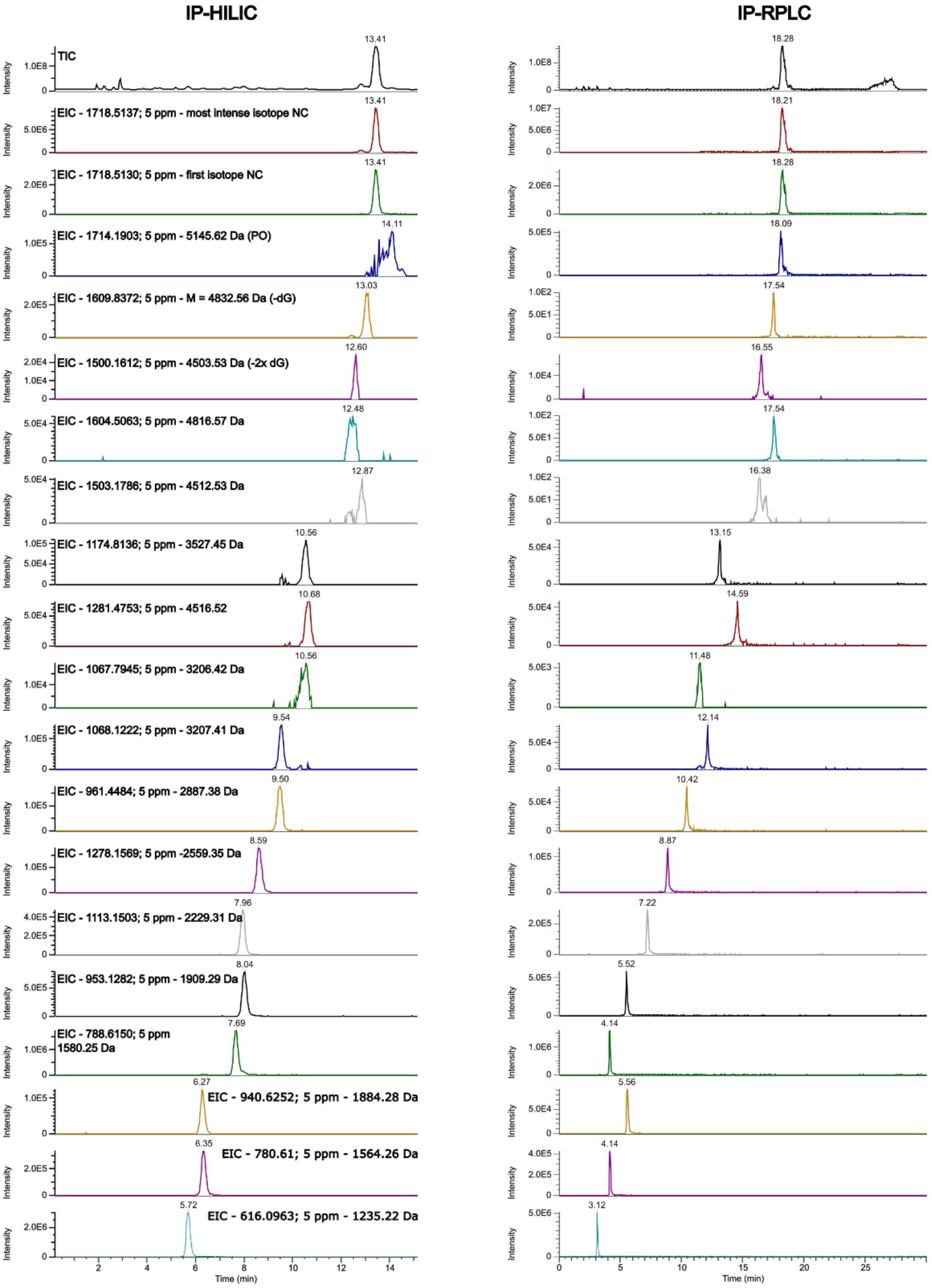

Figure S19: IP-HILIC-UV-MS (left) and IP-RPLC-MS (right) of the mixture of NC and DA-NC. IP-HILIC conditions: concentration TEA in eluent, 25 mM; eluent pH, 6.3; column temperature, 80 °C; linear gradient: 80% B to 66.7% B from 1 to 21 min at 0.2 mL/min; isCID energy, 20 eV. IP-RPLC conditions: concentration TBuAA in eluent, 5 mM; solvent A, ACN-water 10:90 (v/v); solvent B, ACN-water 80:20 (v/v) ; column, BEH C18; linear gradient: 40%B to 60% B from 1 to 21 min; flow rate, 0.2 mL/min; column temperature, 60 °C. UV absorbance detection at 260 nm. Other conditions, see Experimental Section.

Deconvolution of mass spectra obtained of ONs with IP-HILIC was performed using Unidec applying the following settings: charge range, 2 – 5; mass range, 4000 – 8000; Sample mass, every 1 Da; negative mode; peak detection range, 1 Da; peak detection threshold, 0.1. Mass spectra of ONs obtained during IP-HILIC-MS under optimized conditions: Fig. S14, FLP (6793 Da); Fig. S15, N-1 (6449 Da) and DA (6794 Da); Fig. S16, N+1 (7138 Da) and PO (6777 Da); Fig. S17 NC (5162 Da). The additionally detected masses listed in the figures originate from ON adducts.

Deconvolution of mass spectra obtained of the NC test mixture was also performed with UniDec using the same settings as described above. Here, mass spectra of the ONs obtained during IP-HILIC-MS under optimized conditions: Fig. S19: NC (5162 Da); Fig. S20 DA-NC (5163 Da).

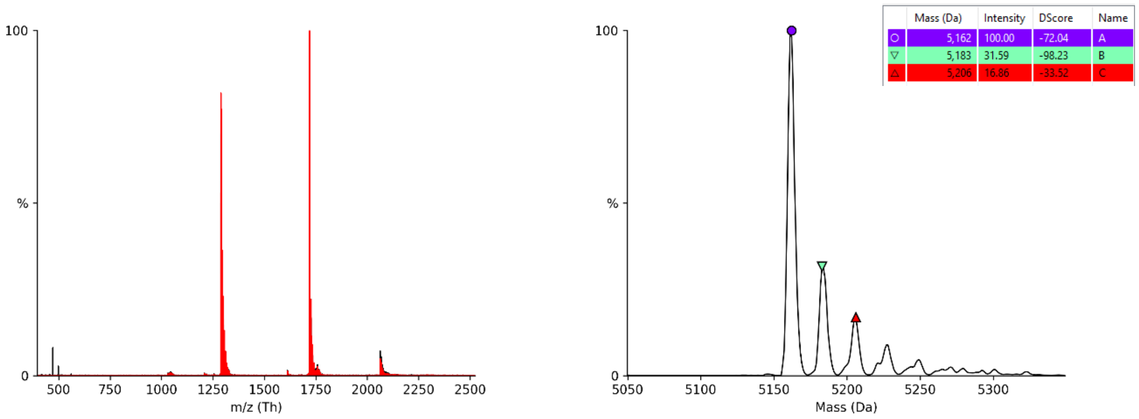

Figure S20: Mass spectrum and deconvoluted mass spectrum of peak eluting at 13.41 min during IP-HILIC-MS of mixture of NC and DA-NC (see Fig. S18). Red indicated m/z window is used for deconvolution.

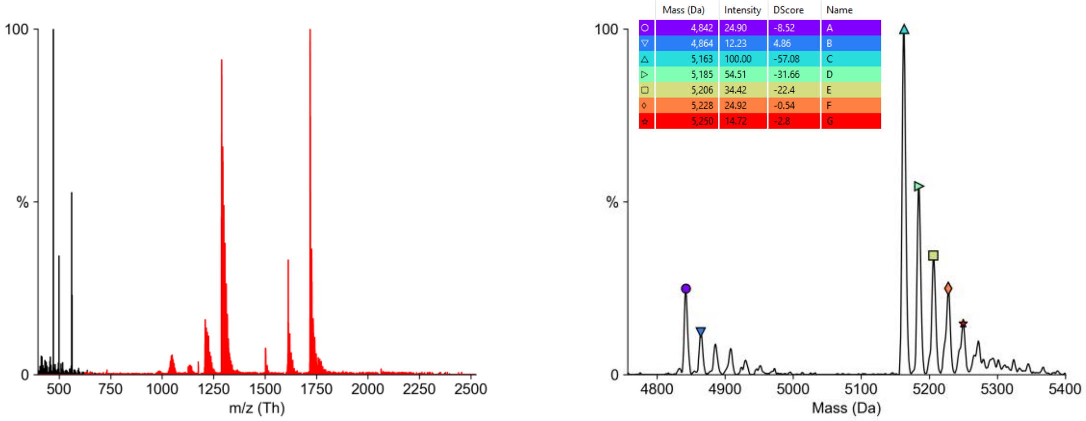

Figure S21: Mass spectrum and deconvoluted mass spectrum of peak eluting at 12.75 min during IP-HILIC-MS of mixture of NC and DA-NC (see Fig. S18). Red indicated m/z window is used for deconvolution.
